# Supplementary material for: Controlling CRISPR-Cas9 with ligand-activated and ligand-deactivated sgRNAs
Source: Nat Commun. 2019 May 9;10:2127. doi: 10.1038/s41467-019-09985-2 (PMC6509140; doi:10.1038/s41467-019-09985-2)
Supplement: Supplementary file 1 — Supplementary Information [file 41467_2019_9985_MOESM1_ESM.pdf]

Supplementary Information to:

Controlling CRISPR-Cas9 with ligand-activated and ligand-deactivated sgRNAs

Kundert *et al*.

SUPPLEMENTARY INFORMATION

Supplementary Notes

Supplementary Note 1 Additional details on the rational design strategies
Supplementary Note 2 Influence of the rational designs on the FACS libraries

Supplementary Tables

Supplementary Table 1 Complete results of the *in vitro* screen of rational designs
Supplementary Table 2 Library sequences screened by FACS
Supplementary Table 3 Ligand-sensitive sgRNAs isolated from FACS screens
Supplementary Table 4 DNA sequences of main constructs
Supplementary Table 5 Detailed results for the *in vitro* DNA cleavage assay in the context of different spacers
Supplementary Table 6 qPCR primers

Supplementary Figures

Supplementary Figure 1 Test of rational designs in *E. coli*
Supplementary Figure 2 Similarities between the rational designs and the FACS libraries
Supplementary Figure 3 Secondary structure predictions suggest mechanisms of ligand sensitivity
Supplementary Figure 4 Mechanistic insights into ligRNA^{-} function
Supplementary Figure 5 ligRNA activity with two promoter strengths
Supplementary Figure 6 ligRNA variants with shifted dynamic ranges
Supplementary Figure 7 Representative gel from the *in vitro* spacer assay
Supplementary Figure 8 Correlation between ligRNA function and the predicted binding free energy of base-pairing between the spacer and the aptamer insert
Supplementary Figure 9 ligRNA function targeting the *E. coli lac* operon
Supplementary Figure 10 ligRNAs function with two different aptamers, four different spacers and three different ligands
Supplementary Figure 11 Thiamine-sensitive ligRNAs
Supplementary Figure 12 Test of ligRNA-mediated target editing in mammalian cell lines
Supplementary Figure 13 Test of ligRNA-mediated gene repression in yeast cells
Supplementary Figure 14 qPCR primer validation

Supplementary References

SUPPLEMENTARY NOTE 1: Additional details on the rational design strategies

Stem replacement (i): Relatively few of the interactions between Cas9 and its sgRNA are specific to the sequence of the sgRNA[1]. Most of the interactions instead involve the RNA sugar-phosphate backbone, suggesting that in these regions the 3D shape of the sgRNA is the primary determinant of Cas9 binding. This hypothesis is supported by mutagenesis data[2]. As the theophylline aptamer adopts a conformation resembling duplex RNA only in the presence of ligand[3], we reasoned that by replacing different portions of different stems with the aptamer, we might create ligand-activated sgRNAs. In some designs, we included sequences on either side of the aptamer following the pattern UUUCCC…, with the intention of discouraging the duplex state.

Induced dimerization (ii): Several aptamers, including the theophylline aptamer[4], can be split and used to dimerize two strands of RNA in the presence of ligand[5]. Given that gRNAs in natural CRISPR systems comprise two strands of RNA (crRNA and tracrRNA) dimerized after a series of maturation steps, we reasoned that it might be possible to create ligand-activated gRNAs by splitting the gRNA into two non-functional halves and using a split-aptamer to artificially control their dimerization into a functional whole.

We applied this strategy to the upper stem, where the natural break between the crRNA and tracrRNA is located. We tested 5 different truncations of the upper stem, with the intent of eliminating any ligand-independent annealing while leaving enough of the upper stem for a functional Cas9/gRNA complex to form. None of the tested designs exhibited any cleavage with or without ligand (**Supplementary Table 1**). Given the lack of detectable activity in *in vitro* conditions that were relatively favorable (controllable concentrations of RNA, ligand, and Cas9; no competing interactions that could be present in cells), we did not pursue this strategy further.

Strand displacement (iii): Riboswitches commonly function by using an aptamer to switch between two conformational states with different base-pairing patterns. In one state, a functional motif is sequestered in an RNA duplex by a complementary strand. In the other state, the functional motif is revealed because the complementary strand base-pairs instead with another strand. This concept has been applied repeatedly to create artificial riboswitches[6-8]. We sought to apply the same concept to create ligand-activated sgRNAs. Specifically, we sought to conditionally sequester the regions of the sgRNA scaffold that are most sensitive to mutation[2], reasoning that these regions would provide the most control over sgRNA function.

The regions we attempted to sequester were the bulge, the nexus, and the “ruler” (our name for the region between the nexus and the hairpin). The bulge forms several sequence-specific interactions with Cas9[1] and also introduces a necessary kink into the upper stem[2]. The nexus forms one sequence-specific interaction with Cas9[1] and a stem that is sensitive to changes in length[2]. The ruler is single-stranded, but sensitive to insertions or deletions, suggesting that the spacing between the nexus and the hairpin is important[2].

We applied two topologies in our strand displacement designs (X is the sequence being sequestered, X’ is a sequence complementary to X, X’’ is a sequence complementary to X’):

X-tetraloop-X’-aptamer-X’’
X-X’’-aptamer-X’

In either case, our intention was that the active state would involve base-pairing between X’ and X’’, the inactive state would involve base-pairing between X’ and X, and the aptamer would drive the transition from one state to the other. The aptamer was always inserted into one of the solvent-exposed stems: the upper stem, the nexus, or the hairpin. The particular sequences of X’ and X’’ were chosen to maintain all important features of the sgRNA as characterized by Briner *et al.*[2], and depended on where the aptamer was inserted relative to the target sequence (**Supplementary Table 1**).

Some of the above designs were constitutively (i.e., both in the presence and in the absence of theophylline) either active or inactive (**Supplementary Table 1**). For the constitutively active designs, we attempted to weaken the active state by making complementary mutations in X’ and X’’ that would introduce wobble base-pairs or mismatches between X’ and X. For the constitutively inactive designs, we attempted to weaken the inactive state by making mutations in X’’ that would introduce wobble base-pairs of mismatches between X’ and X’’.

SUPPLEMENTARY NOTE 2: Influence of the rational designs on the FACS libraries

Although the rational designs only had weak ligand-sensitivity in a CRISPRi-based assay in *E. coli* (**Supplementary Figure 1**), they informed the design of the libraries used in the FACS screens in four key aspects:

(i) Insertion sites: We used the same aptamer insertion sites as tested in the rational designs for the library designs, as we were able to find rational designs that were functional *in vitro* for all three sites (**Figure 1**, **Supplementary Table 1**). **Supplementary Figure 2** shows a comparison between the rational and library designs for each insertion site.

(ii) Strand displacement mechanism: Since the strand displacement was the most effective rational design strategy (**Supplementary Table 1**), we built libraries consistent with sequestering the same motifs. In particular, the upper stem libraries (**Supplementary Table 2**: #1–#6) included the bulge, and the second batch of hairpin libraries (**Supplementary Table 2**: #29, #30) included part of the nexus. Note that secondary structure predictions of ligRNA^{+}, which was isolated from library #29, are consistent with a strand displacement mechanism involving base-pairing between the aptamer (**Supplementary Figure 3**).

(iii) Nexus libraries: The nexus stem is short and sensitive to mutation, both properties that make the strand displacement strategy difficult to implement. Instead, the rational designs with the aptamer inserted into the nexus were designed using the stem replacement strategy. The designs included 0–5 nt linkers on either side of the aptamer, but only those with 2–5 nt linkers exhibited any *in vitro* cleavage activity (**Supplementary Table 1**). The nexus libraries (**Supplementary Table 2**, #7–#22) expanded on this approach, with randomized 2–5 nt linkers on either side of the aptamer. In addition, since inserting the aptamer into the nexus consistently produced sgRNAs that were deactivated (instead of activated) by theophylline, we screened all of the libraries for both ligand-activated and -deactivated ligRNAs.

(iv) Induced dimerization: Considering that the induced dimerization strategy was not successful *in vitro*, we did not pursue it in *E. coli*.

Supplementary Table 1

#	Strategy	Domain	Cleavage (%)				N	Active?	Sequence
			apo	*holo*	Δ	σ			
1	Positive Control		87	87	0	5	13		GGGGCCACUAGGGACAGGAUGUUUUAGAGCUAGAAAUAGCAAGUUAAAAUAAGGCUAGUCCGUUAUCAACUUGAAAAAGUGGCACCGAGUCGGUGCUUUUUU
2	Negative Control		0	0	-0	0	13		GGGGCCACUAGGGACAGGAUGUUUUAGAGCUAGAAAUAGCAAGUUAAAAUAACCCUAGUCCGUUAUCAACUUGAAAAAGUGGCACCGAGUCGGUGCUUUUUU
3	Stem Replacement	Upper Stem	32	47	15	15	4		GGGGCCACUAGGGACAGGAUGUUUUAGA------------------AUACCAGCCGAAAGGCCCUUGGCAG------------------AAGUUAAAAUAAGGCUAGUCCGUUAUCAACUUGAAAAAGUGGCACCGAGUCGGUGCUUUUUU
4	Stem Replacement	Upper Stem	100	100	0		1		GGGGCCACUAGGGACAGGAUGUUUUAGA----U-------------AUACCAGCCGAAAGGCCCUUGGCAG-------------U----AAGUUAAAAUAAGGCUAGUCCGUUAUCAACUUGAAAAAGUGGCACCGAGUCGGUGCUUUUUU
5	Stem Replacement	Upper Stem	66	81	15		2	✓	GGGGCCACUAGGGACAGGAUGUUUUAGA----UU------------AUACCAGCCGAAAGGCCCUUGGCAG------------UU----AAGUUAAAAUAAGGCUAGUCCGUUAUCAACUUGAAAAAGUGGCACCGAGUCGGUGCUUUUUU
6	Stem Replacement	Upper Stem	40	46	6	5	3		GGGGCCACUAGGGACAGGAUGUUUUAGA----UUU-----------AUACCAGCCGAAAGGCCCUUGGCAG-----------UUU----AAGUUAAAAUAAGGCUAGUCCGUUAUCAACUUGAAAAAGUGGCACCGAGUCGGUGCUUUUUU
7	Stem Replacement	Upper Stem	61	79	18		2	✓	GGGGCCACUAGGGACAGGAUGUUUUAGAG-----------------AUACCAGCCGAAAGGCCCUUGGCAG-----------------CAAGUUAAAAUAAGGCUAGUCCGUUAUCAACUUGAAAAAGUGGCACCGAGUCGGUGCUUUUUU
8	Stem Replacement	Upper Stem	76	86	10		2		GGGGCCACUAGGGACAGGAUGUUUUAGAG---U-------------AUACCAGCCGAAAGGCCCUUGGCAG-------------U---CAAGUUAAAAUAAGGCUAGUCCGUUAUCAACUUGAAAAAGUGGCACCGAGUCGGUGCUUUUUU
9	Stem Replacement	Upper Stem	75	78	3		2		GGGGCCACUAGGGACAGGAUGUUUUAGAG---UU------------AUACCAGCCGAAAGGCCCUUGGCAG------------UU---CAAGUUAAAAUAAGGCUAGUCCGUUAUCAACUUGAAAAAGUGGCACCGAGUCGGUGCUUUUUU
10	Stem Replacement	Upper Stem	63	66	3		2		GGGGCCACUAGGGACAGGAUGUUUUAGAG---UUU-----------AUACCAGCCGAAAGGCCCUUGGCAG-----------UUU---CAAGUUAAAAUAAGGCUAGUCCGUUAUCAACUUGAAAAAGUGGCACCGAGUCGGUGCUUUUUU
11	Stem Replacement	Upper Stem	84	83	-0		2		GGGGCCACUAGGGACAGGAUGUUUUAGAGC--UU------------AUACCAGCCGAAAGGCCCUUGGCAG------------UU--GCAAGUUAAAAUAAGGCUAGUCCGUUAUCAACUUGAAAAAGUGGCACCGAGUCGGUGCUUUUUU
12	Stem Replacement	Upper Stem	51	53	1		2		GGGGCCACUAGGGACAGGAUGUUUUAGAGC--UUUCCC--------AUACCAGCCGAAAGGCCCUUGGCAG--------UUUCCC--GCAAGUUAAAAUAAGGCUAGUCCGUUAUCAACUUGAAAAAGUGGCACCGAGUCGGUGCUUUUUU
13	Stem Replacement	Upper Stem	63	66	4		2		GGGGCCACUAGGGACAGGAUGUUUUAGAGC--UUUCCCUUUC----AUACCAGCCGAAAGGCCCUUGGCAG----UUUCCCUUUC--GCAAGUUAAAAUAAGGCUAGUCCGUUAUCAACUUGAAAAAGUGGCACCGAGUCGGUGCUUUUUU
14	Stem Replacement	Upper Stem	36	31	-6		1		GGGGCCACUAGGGACAGGAUGUUUUAGAGC--UUUCCCUUUCCCUUAUACCAGCCGAAAGGCCCUUGGCAGUUUCCCUUUCCCUU--GCAAGUUAAAAUAAGGCUAGUCCGUUAUCAACUUGAAAAAGUGGCACCGAGUCGGUGCUUUUUU
15	Stem Replacement	Upper Stem	97	95	-2		2		GGGGCCACUAGGGACAGGAUGUUUUAGAGCUAUU------------AUACCAGCCGAAAGGCCCUUGGCAG------------UUUAGCAAGUUAAAAUAAGGCUAGUCCGUUAUCAACUUGAAAAAGUGGCACCGAGUCGGUGCUUUUUU
16	Stem Replacement	Upper Stem	72	72	-0		2		GGGGCCACUAGGGACAGGAUGUUUUAGAGCUAUUUCCC--------AUACCAGCCGAAAGGCCCUUGGCAG--------UUUCCCUAGCAAGUUAAAAUAAGGCUAGUCCGUUAUCAACUUGAAAAAGUGGCACCGAGUCGGUGCUUUUUU
17	Stem Replacement	Upper Stem	96	97	1		2		GGGGCCACUAGGGACAGGAUGUUUUAGAGCUAUUUCCCUUUC----AUACCAGCCGAAAGGCCCUUGGCAG----UUUCCCUUUCUAGCAAGUUAAAAUAAGGCUAGUCCGUUAUCAACUUGAAAAAGUGGCACCGAGUCGGUGCUUUUUU
18	Stem Replacement	Upper Stem	93	87	-5		2		GGGGCCACUAGGGACAGGAUGUUUUAGAGCUAUUUCCCUUUCCCUUAUACCAGCCGAAAGGCCCUUGGCAGUUUCCCUUUCCCUUUAGCAAGUUAAAAUAAGGCUAGUCCGUUAUCAACUUGAAAAAGUGGCACCGAGUCGGUGCUUUUUU
19	Stem Replacement	Lower Stem	0	0	0		1		GGGGCCACUAGGGACAGGAUGUUUU---------------------AUACCAGCCGAAAGGCCCUUGGCAG-----------------------AAAAUAAGGCUAGUCCGUUAUCAACUUGAAAAAGUGGCACCGAGUCGGUGCUUUUUU
20	Stem Replacement	Lower Stem	0	0	0		1		GGGGCCACUAGGGACAGGAUGUUUUA--------------------AUACCAGCCGAAAGGCCCUUGGCAG----------------------UAAAAUAAGGCUAGUCCGUUAUCAACUUGAAAAAGUGGCACCGAGUCGGUGCUUUUUU
21	Stem Replacement	Lower Stem	0	0	0		1		GGGGCCACUAGGGACAGGAUGUUUUA------UU------------AUACCAGCCGAAAGGCCCUUGGCAG------------UU--------UAAAAUAAGGCUAGUCCGUUAUCAACUUGAAAAAGUGGCACCGAGUCGGUGCUUUUUU
22	Stem Replacement	Lower Stem	0	0	0		1		GGGGCCACUAGGGACAGGAUGUUUUA------UUUC----------AUACCAGCCGAAAGGCCCUUGGCAG----------UUUC--------UAAAAUAAGGCUAGUCCGUUAUCAACUUGAAAAAGUGGCACCGAGUCGGUGCUUUUUU
23	Stem Replacement	Lower Stem	0	0	0		1		GGGGCCACUAGGGACAGGAUGUUUUA------UUUCCC--------AUACCAGCCGAAAGGCCCUUGGCAG--------UUUCCC--------UAAAAUAAGGCUAGUCCGUUAUCAACUUGAAAAAGUGGCACCGAGUCGGUGCUUUUUU
24	Strand displacement	Upper Stem	14	70	56	19	4	✓	GGGGCCACUAGGGACAGGAUGUUUUA----------------ACUUAUACCAGCCGAAAGGCCCUUGGCAGAA-GU-------------AAGUUAAAAUAAGGCUAGUCCGUUAUCAACUUGAAAAAGUGGCACCGAGUCGGUGCUUUUUU
25	Strand displacement	Upper Stem	7	73	67	7	3	✓	GGGGCCACUAGGGACAGGAUGUUUUA----------------ACUUAUACCAGCCGAAAGGCCCUUGGCAGAG-GU-------------AAGUUAAAAUAAGGCUAGUCCGUUAUCAACUUGAAAAAGUGGCACCGAGUCGGUGCUUUUUU
26	Strand displacement	Upper Stem	2	16	13		2		GGGGCCACUAGGGACAGGAUGUUUUA----------------ACUUAUACCAGCCGAAAGGCCCUUGGCAGAC-GU-------------AAGUUAAAAUAAGGCUAGUCCGUUAUCAACUUGAAAAAGUGGCACCGAGUCGGUGCUUUUUU
27	Strand displacement	Upper Stem	6	35	29		2	✓	GGGGCCACUAGGGACAGGAUGUUUUA----------------ACUUAUACCAGCCGAAAGGCCCUUGGCAGAAAGU-------------AAGUUAAAAUAAGGCUAGUCCGUUAUCAACUUGAAAAAGUGGCACCGAGUCGGUGCUUUUUU
28	Strand displacement	Upper Stem	1	1	1		1		GGGGCCACUAGGGACAGGAUGUUUUA----------------ACUUAUACCAGCCGAAAGGCCCUUGGCAGGG-GU-------------AAGUUAAAAUAAGGCUAGUCCGUUAUCAACUUGAAAAAGUGGCACCGAGUCGGUGCUUUUUU
29	Strand displacement	Lower Stem	75	75	-0	3	3		GGGGCCACUAGGGACAGGAUAGCCUUGA------------------AUACCAGCCGAAAGGCCCUUGGCAG------------------AAGUAAGGCUAAGGCUAGUCCGUUAUCAACUUGAAAAAGUGGCACCGAGUCGGUGCUUUUUU
30	Strand displacement	Lower Stem	76	81	5		2		GGGGCCACUAGGGACAGGAUAGCCUUGA------------------AUACCAGCCGAAAGGCCCUUGGCAG------------------AAGUAGGGCUAAGGCUAGUCCGUUAUCAACUUGAAAAAGUGGCACCGAGUCGGUGCUUUUUU
31	Strand displacement	Lower Stem	2	5	3		1		GGGGCCACUAGGGACAGGAUAGCCUUGA------------------AUACCAGCCGAAAGGCCCUUGGCAG------------------AAGUAACGCUAAGGCUAGUCCGUUAUCAACUUGAAAAAGUGGCACCGAGUCGGUGCUUUUUU
32	Strand displacement	Lower Stem	0	0	0		1		GGGGCCACUAGGGACAGGAUAGCCUUGA------------------AUACCAGCCGAAAGGCCCUUGGCAG-----------------AAGUAAGAGCUAAGGCUAGUCCGUUAUCAACUUGAAAAAGUGGCACCGAGUCGGUGCUUUUUU
33	Strand displacement	Upper Stem	74	77	3	7	3		GGGGCCACUAGGGACAGGAUGUUUUAGA--------UCGU------AUACCAGCCGAAAGGCCCUUGGCAG------ACGA--------AAGUUAAAAUAAGGCUAGUCCGUUAUCAACUUGAAAAAGUGGCACCGAGUCGGUGCUUUUUU
34	Strand displacement	Upper Stem	80	80	-0	0	3		GGGGCCACUAGGGACAGGAUGUUUUAGA--------UCGUUAA---AUACCAGCCGAAAGGCCCUUGGCAG---UUAACGA--------AAGUUAAAAUAAGGCUAGUCCGUUAUCAACUUGAAAAAGUGGCACCGAGUCGGUGCUUUUUU
35	Strand displacement	Upper Stem	47	52	6	6	3		GGGGCCACUAGGGACAGGAUGUUUUAGA--------UCGUUAAAAUAUACCAGCCGAAAGGCCCUUGGCAGAUUUUAACGA--------AAGUUAAAAUAAGGCUAGUCCGUUAUCAACUUGAAAAAGUGGCACCGAGUCGGUGCUUUUUU
36	Strand displacement	Upper Stem	91	94	3		2		GGGGCCACUAGGGACAGGAUGUUUUAGA--------UCGAU-----AUACCAGCCGAAAGGCCCUUGGCAG------ACGA--------AAGUUAAAAUAAGGCUAGUCCGUUAUCAACUUGAAAAAGUGGCACCGAGUCGGUGCUUUUUU
37	Strand displacement	Upper Stem	94	94	-0		2		GGGGCCACUAGGGACAGGAUGUUUUAGA--------UCGUU-----AUACCAGCCGAAAGGCCCUUGGCAG-----AACGA--------AAGUUAAAAUAAGGCUAGUCCGUUAUCAACUUGAAAAAGUGGCACCGAGUCGGUGCUUUUUU
38	Strand displacement	Upper Stem	95	95	-0		2		GGGGCCACUAGGGACAGGAUGUUUUAGA--------UCGCU-----AUACCAGCCGAAAGGCCCUUGGCAG-----AACGA--------AAGUUAAAAUAAGGCUAGUCCGUUAUCAACUUGAAAAAGUGGCACCGAGUCGGUGCUUUUUU
39	Strand displacement	Upper Stem	94	95	1		2		GGGGCCACUAGGGACAGGAUGUUUUAGA--------UCGAUU----AUACCAGCCGAAAGGCCCUUGGCAG-----AACGA--------AAGUUAAAAUAAGGCUAGUCCGUUAUCAACUUGAAAAAGUGGCACCGAGUCGGUGCUUUUUU
40	Strand displacement	Upper Stem	94	94	-0		2		GGGGCCACUAGGGACAGGAUGUUUUAGA--------UCGUUA----AUACCAGCCGAAAGGCCCUUGGCAG----UAACGA--------AAGUUAAAAUAAGGCUAGUCCGUUAUCAACUUGAAAAAGUGGCACCGAGUCGGUGCUUUUUU
41	Strand displacement	Upper Stem	94	94	0		2		GGGGCCACUAGGGACAGGAUGUUUUAGA--------UCGUUG----AUACCAGCCGAAAGGCCCUUGGCAG----UAACGA--------AAGUUAAAAUAAGGCUAGUCCGUUAUCAACUUGAAAAAGUGGCACCGAGUCGGUGCUUUUUU
42	Strand displacement	Upper Stem	95	94	-1		2		GGGGCCACUAGGGACAGGAUGUUUUAGA--------UCGCUA----AUACCAGCCGAAAGGCCCUUGGCAG----UAACGA--------AAGUUAAAAUAAGGCUAGUCCGUUAUCAACUUGAAAAAGUGGCACCGAGUCGGUGCUUUUUU
43	Strand displacement	Upper Stem	95	95	-0		2		GGGGCCACUAGGGACAGGAUGUUUUAGA--------UCGUAUA---AUACCAGCCGAAAGGCCCUUGGCAG----UAACGA--------AAGUUAAAAUAAGGCUAGUCCGUUAUCAACUUGAAAAAGUGGCACCGAGUCGGUGCUUUUUU
44	Strand displacement	Upper Stem	46	57	11		2		GGGGCCACUAGGGACAGGAUGUUUUAGA--------UCGCUAA---AUACCAGCCGAAAGGCCCUUGGCAG---UUAACGA--------AAGUUAAAAUAAGGCUAGUCCGUUAUCAACUUGAAAAAGUGGCACCGAGUCGGUGCUUUUUU
45	Strand displacement	Upper Stem	93	93	0		2		GGGGCCACUAGGGACAGGAUGUUUUAGA--------UCGUUAAA--AUACCAGCCGAAAGGCCCUUGGCAG--UUUAACGA--------AAGUUAAAAUAAGGCUAGUCCGUUAUCAACUUGAAAAAGUGGCACCGAGUCGGUGCUUUUUU
46	Strand displacement	Upper Stem	14	38	24		2	✓	GGGGCCACUAGGGACAGGAUGUUUUAGA--------UCGCUAAA--AUACCAGCCGAAAGGCCCUUGGCAG--UUUAACGA--------AAGUUAAAAUAAGGCUAGUCCGUUAUCAACUUGAAAAAGUGGCACCGAGUCGGUGCUUUUUU
47	Strand displacement	Upper Stem	67	79	13		1		GGGGCCACUAGGGACAGGAUGUUUUAGA--------UCGUUGAA--AUACCAGCCGAAAGGCCCUUGGCAG--UUUAACGA--------AAGUUAAAAUAAGGCUAGUCCGUUAUCAACUUGAAAAAGUGGCACCGAGUCGGUGCUUUUUU
48	Strand displacement	Lower Stem	0	0	0	0	3		GGGGCCACUAGGGACAGGAUUCGGCUGA------------------AUACCAGCCGAAAGGCCCUUGGCAG------------------AAGUAGCCGAAAGGCUAGUCCGUUAUCAACUUGAAAAAGUGGCACCGAGUCGGUGCUUUUUU
49	Strand displacement	Lower Stem	70	72	2	3	3		GGGGCCACUAGGGACAGGAUGUUAUCGA------------------AUACCAGCCGAAAGGCCCUUGGCAG------------------AAGUGAUAACAAGGCUAGUCCGUUAUCAACUUGAAAAAGUGGCACCGAGUCGGUGCUUUUUU
50	Strand displacement	Lower Stem	65	69	4		2		GGGGCCACUAGGGACAGGAUGUUGUCGA------------------AUACCAGCCGAAAGGCCCUUGGCAG------------------AAGUGAUAACAAGGCUAGUCCGUUAUCAACUUGAAAAAGUGGCACCGAGUCGGUGCUUUUUU
51	Strand displacement	Lower Stem	27	32	4		2		GGGGCCACUAGGGACAGGAUGGUAUCGA------------------AUACCAGCCGAAAGGCCCUUGGCAG------------------AAGUGAUACCAAGGCUAGUCCGUUAUCAACUUGAAAAAGUGGCACCGAGUCGGUGCUUUUUU
52	Strand displacement	Lower Stem	9	11	2		1		GGGGCCACUAGGGACAGGAUGCUAUCGA------------------AUACCAGCCGAAAGGCCCUUGGCAG------------------AAGUGAUAACAAGGCUAGUCCGUUAUCAACUUGAAAAAGUGGCACCGAGUCGGUGCUUUUUU
53	Strand displacement	Lower Stem	8	13	5		1		GGGGCCACUAGGGACAGGAUGUUAAUCGA-----------------AUACCAGCCGAAAGGCCCUUGGCAG------------------AAGUGAUAACAAGGCUAGUCCGUUAUCAACUUGAAAAAGUGGCACCGAGUCGGUGCUUUUUU
54	Induced Dimerization	Upper Stem	0	0	0		1		GGGGCCACUAGGGACAGGAUGUUUUAGA------------------AUACCAGCC GGCCCUUGGCAG------------------AAGUUAAAAUAAGGCUAGUCCGUUAUCAACUUGAAAAAGUGGCACCGAGUCGGUGCUUUUUU
55	Induced Dimerization	Upper Stem	0	0	0		2		GGGGCCACUAGGGACAGGAUGUUUUAGAG-----------------AUACCAGCC GGCCCUUGGCAG-----------------CAAGUUAAAAUAAGGCUAGUCCGUUAUCAACUUGAAAAAGUGGCACCGAGUCGGUGCUUUUUU
56	Induced Dimerization	Upper Stem	0	0	0		2		GGGGCCACUAGGGACAGGAUGUUUUAGAGC----------------AUACCAGCC GGCCCUUGGCAG----------------GCAAGUUAAAAUAAGGCUAGUCCGUUAUCAACUUGAAAAAGUGGCACCGAGUCGGUGCUUUUUU
57	Induced Dimerization	Upper Stem	0	0	0		2		GGGGCCACUAGGGACAGGAUGUUUUAGAGCU---------------AUACCAGCC GGCCCUUGGCAG---------------AGCAAGUUAAAAUAAGGCUAGUCCGUUAUCAACUUGAAAAAGUGGCACCGAGUCGGUGCUUUUUU
58	Induced Dimerization	Upper Stem	0	0	0		2		GGGGCCACUAGGGACAGGAUGUUUUAGAGCUA--------------AUACCAGCC GGCCCUUGGCAG--------------UAGCAAGUUAAAAUAAGGCUAGUCCGUUAUCAACUUGAAAAAGUGGCACCGAGUCGGUGCUUUUUU
59	Stem Replacement	Nexus	0	0	0		1		GGGGCCACUAGGGACAGGAUGUUUUAGAGCUAGAAAUAGCAAGUUAAAAUAA-----AUACCAGCCGAAAGGCCCUUGGCAG-----GUUAUCAACUUGAAAAAGUGGCACCGAGUCGGUGCUUUUUU
60	Stem Replacement	Nexus	0	0	0		1		GGGGCCACUAGGGACAGGAUGUUUUAGAGCUAGAAAUAGCAAGUUAAAAUAAG----AUACCAGCCGAAAGGCCCUUGGCAG----CGUUAUCAACUUGAAAAAGUGGCACCGAGUCGGUGCUUUUUU
61	Stem Replacement	Nexus	43	10	-32	8	5	✓	GGGGCCACUAGGGACAGGAUGUUUUAGAGCUAGAAAUAGCAAGUUAAAAUAAGG---AUACCAGCCGAAAGGCCCUUGGCAG---CCGUUAUCAACUUGAAAAAGUGGCACCGAGUCGGUGCUUUUUU
62	Stem Replacement	Nexus	66	60	-6		1		GGGGCCACUAGGGACAGGAUGUUUUAGAGCUAGAAAUAGCAAGUUAAAAUAAGG---AUACCAGCCGAAAGGCCCUUGGCAG--UCCGUUAUCAACUUGAAAAAGUGGCACCGAGUCGGUGCUUUUUU
63	Stem Replacement	Nexus	5	4	-1		1		GGGGCCACUAGGGACAGGAUGUUUUAGAGCUAGAAAUAGCAAGUUAAAAUAAGGC--AUACCAGCCGAAAGGCCCUUGGCAG-GUCCGUUAUCAACUUGAAAAAGUGGCACCGAGUCGGUGCUUUUUU
64	Stem Replacement	Nexus	95	98	3		1		GGGGCCACUAGGGACAGGAUGUUUUAGAGCUAGAAAUAGCAAGUUAAAAUAAGGU--AUACCAGCCGAAAGGCCCUUGGCAG--UCCGUUAUCAACUUGAAAAAGUGGCACCGAGUCGGUGCUUUUUU
65	Stem Replacement	Nexus	84	70	-14		1		GGGGCCACUAGGGACAGGAUGUUUUAGAGCUAGAAAUAGCAAGUUAAAAUAAGGUU-AUACCAGCCGAAAGGCCCUUGGCAG-UUCCGUUAUCAACUUGAAAAAGUGGCACCGAGUCGGUGCUUUUUU
66	Stem Replacement	Nexus	34	26	-8		1		GGGGCCACUAGGGACAGGAUGUUUUAGAGCUAGAAAUAGCAAGUUAAAAUAAGGUUUAUACCAGCCGAAAGGCCCUUGGCAGUUUCCGUUAUCAACUUGAAAAAGUGGCACCGAGUCGGUGCUUUUUU
67	Stem Replacement	Hairpin	100	100	0		1		GGGGCCACUAGGGACAGGAUGUUUUAGAGCUAGAAAUAGCAAGUUAAAAUAAGGCUAGUCCGUUAUCAACUUGAAAAAGU----------------AUACCAGCCGAAAGGCCCUUGGCAG-----------------------UUUUUU
68	Stem Replacement	Hairpin	53	56	3	9	3		GGGGCCACUAGGGACAGGAUGUUUUAGAGCUAGAAAUAGCAAGUUAAAAUAAGGCUAGUCCGUUAUCAACUUGAAAAAGUG---------------AUACCAGCCGAAAGGCCCUUGGCAG-----------------------UUUUUU
69	Stem Replacement	Hairpin	100	100	0		1		GGGGCCACUAGGGACAGGAUGUUUUAGAGCUAGAAAUAGCAAGUUAAAAUAAGGCUAGUCCGUUAUCAACUUGAAAAAGUGGCACCGAGUCGGUGCAUACCAGCCGAAAGGCCCUUGGCAG-----------------------UUUUUU
70	Stem Replacement	Hairpin	60	70	10	5	3		GGGGCCACUAGGGACAGGAUGUUUUAGAGCUAGAAAUAGCAAGUUAAAAUAAGGCUAGUCCGUUAUCA----------------------------AUACCAGCCGAAAGGCCCUUGGCAG-------GGCACCGAGUCGGUGCUUUUUU
71	Strand displacement	Hairpin	63	68	5	0	3		GGGGCCACUAGGGACAGGAUGUUUUAGAGCUAGAAAUAGCAAGUUAAAAUAAGGCUAGUCCGUUAUCA------------------------AUCAAUACCAGCCGAAAGGCCCUUGGCAGUGAU---GGCACCGAGUCGGUGCUUUUUU
72	Strand displacement	Hairpin	81	84	4		2		GGGGCCACUAGGGACAGGAUGUUUUAGAGCUAGAAAUAGCAAGUUAAAAUAAGGCUAGUCCGUUAUCA-----------------------UAUCAAUACCAGCCGAAAGGCCCUUGGCAGUGAUA--GGCACCGAGUCGGUGCUUUUUU
73	Strand displacement	Hairpin	85	89	3		2		GGGGCCACUAGGGACAGGAUGUUUUAGAGCUAGAAAUAGCAAGUUAAAAUAAGGCUAGUCCGUUAUCA-----------------------UGUCAAUACCAGCCGAAAGGCCCUUGGCAGUGAUA--GGCACCGAGUCGGUGCUUUUUU
74	Strand displacement	Hairpin	90	89	-1		2		GGGGCCACUAGGGACAGGAUGUUUUAGAGCUAGAAAUAGCAAGUUAAAAUAAGGCUAGUCCGUUAUCA----------------------UUAUCAAUACCAGCCGAAAGGCCCUUGGCAGUGAUAA-GGCACCGAGUCGGUGCUUUUUU
75	Strand displacement	Hairpin	94	95	1		2		GGGGCCACUAGGGACAGGAUGUUUUAGAGCUAGAAAUAGCAAGUUAAAAUAAGGCUAGUCCGUUAUCA----------------------UUGUCAAUACCAGCCGAAAGGCCCUUGGCAGUGAUAA-GGCACCGAGUCGGUGCUUUUUU
76	Strand displacement	Hairpin	45	52	6		2		GGGGCCACUAGGGACAGGAUGUUUUAGAGCUAGAAAUAGCAAGUUAAAAUAAGGCUAGUCCGUUAUCA--------------------AA-CG---AUACCAGCCGAAAGGCCCUUGGCAG---CGUUGGCACCGAGUCGGUGCUUUUUU
77	Strand displacement	Hairpin	73	80	7		2		GGGGCCACUAGGGACAGGAUGUUUUAGAGCUAGAAAUAGCAAGUUAAAAUAAGGCUAGUCCGUUAUCA--------------------AA-AG---AUACCAGCCGAAAGGCCCUUGGCAG---CUUUGGCACCGAGUCGGUGCUUUUUU
78	Strand displacement	Hairpin	21	34	13		1		GGGGCCACUAGGGACAGGAUGUUUUAGAGCUAGAAAUAGCAAGUUAAAAUAAGGCUAGUCCGUUAUCA--------------------AA-UG---AUACCAGCCGAAAGGCCCUUGGCAG---CAUUGGCACCGAGUCGGUGCUUUUUU
79	Strand displacement	Hairpin	90	92	2		2		GGGGCCACUAGGGACAGGAUGUUUUAGAGCUAGAAAUAGCAAGUUAAAAUAAGGCUAGUCCGUUAUCA--------------------AAACG---AUACCAGCCGAAAGGCCCUUGGCAG--CGUUUGGCACCGAGUCGGUGCUUUUUU
80	Strand displacement	Hairpin	3	9	6	4	4		GGGGCCACUAGGGACAGGAUGUUUUAGAGCUAGAAAUAGCAAGUUAAAAUAAGGCUAGUCCGUUAUCA--------------------AA-CGG--AUACCAGCCGAAAGGCCCUUGGCAG--CCGUUGGCACCGAGUCGGUGCUUUUUU
81	Strand displacement	Hairpin	64	80	16		2	✓	GGGGCCACUAGGGACAGGAUGUUUUAGAGCUAGAAAUAGCAAGUUAAAAUAAGGCUAGUCCGUUAUCA--------------------AA-AGG--AUACCAGCCGAAAGGCCCUUGGCAG--CCUUUGGCACCGAGUCGGUGCUUUUUU
82	Strand displacement	Hairpin	13	30	17		1	✓	GGGGCCACUAGGGACAGGAUGUUUUAGAGCUAGAAAUAGCAAGUUAAAAUAAGGCUAGUCCGUUAUCA--------------------AA-UGG--AUACCAGCCGAAAGGCCCUUGGCAG--CCAUUGGCACCGAGUCGGUGCUUUUUU
83	Strand displacement	Hairpin	87	90	4		2		GGGGCCACUAGGGACAGGAUGUUUUAGAGCUAGAAAUAGCAAGUUAAAAUAAGGCUAGUCCGUUAUCA--------------------AAGCGG--AUACCAGCCGAAAGGCCCUUGGCAG-CCGCUUGGCACCGAGUCGGUGCUUUUUU
84	Strand displacement	Hairpin	82	79	-3		2		GGGGCCACUAGGGACAGGAUGUUUUAGAGCUAGAAAUAGCAAGUUAAAAUAAGGCUAGUCCGUUAUCA--------------------AA-CGGA-AUACCAGCCGAAAGGCCCUUGGCAG-UCCGUUGGCACCGAGUCGGUGCUUUUUU
85	Strand displacement	Hairpin	8	28	20	12	4	✓	GGGGCCACUAGGGACAGGAUGUUUUAGAGCUAGAAAUAGCAAGUUAAAAUAAGGCUAGUCCGUUAUCA--------------------AA-CGGACAUACCAGCCGAAAGGCCCUUGGCAGGUCCGUUGGCACCGAGUCGGUGCUUUUUU
86	Strand displacement	Hairpin	95	94	-1		2		GGGGCCACUAGGGACAGGAUGUUUUAGAGCUAGAAAUAGCAAGUUAAAAUAAGGCUAGUCCGUUAUCA--------------------AA-UGGACAUACCAGCCGAAAGGCCCUUGGCAGGUCCAUUGGCACCGAGUCGGUGCUUUUUU

Supplementary Table 1: Complete results of the *in vitro* screen of rational designs. #: Design number. Strategy: The mechanism by which the design was intended to work. "Stem Replacement" means a stem in the sgRNA was replaced by the aptamer (in some cases including a linker), with the expectation that ligand binding to the aptamer would stabilize the stem to adopt a functional sgRNA conformation. "Induced dimerization" means the sgRNA was split in half, with each half containing part of the aptamer, in the hope that the two halves would dimerize in the presence of ligand. "Strand displacement" means that strands were designed to base pair in two ways – one maintaining the wildtype sgRNA functional conformation and the other adopting an alternative conformation – and that ligand binding to the aptamer would stabilize the functional conformation. Domain: The domain in the sgRNA (as defined in **Figure 1b** in the main text) into which the aptamer was inserted. Cleavage: The percent of DNA that was cleaved by a design in the *in vitro* assay. The *apo* and *holo* columns refer to the cleavage with and without theophylline, respectively, Δ is the difference between these values, and σ is the standard deviation of the Δ values for designs with three or more replicates. All percentages are averages of any replicates and are rounded to the nearest integer. N: The number of replicates for each design. Active: We denote a design as "active" if it exhibited a >15% change in cleavage in response to ligand. Sequence: The sequence of the design, including the AAVS spacer used in this assay (**Supplementary Table 4**). Sequences are aligned and color coded by sgRNA domain as in **Figure 1b** (grey: spacer; blue: upper stem; navy: nexus; teal: hairpin; purple: aptamer; orange: other regions).

Supplementary Table 2

#	Domain	Size	Sequence
1	Upper Stem	4^{10}	GTTTTANNNN--ATACCAGCCGAAAGGCCCTTGGCAG--NNNNNNTAAAATAAGGCTAGTCCGTTATCAACTTGAAAAAGTGGCACCGAGTCGGTGCTTTTTT
2	Upper Stem	4^{11}	GTTTTANNNN--ATACCAGCCGAAAGGCCCTTGGCAG-NNNNNNNTAAAATAAGGCTAGTCCGTTATCAACTTGAAAAAGTGGCACCGAGTCGGTGCTTTTTT
3	Upper Stem	4^{12}	GTTTTANNNN--ATACCAGCCGAAAGGCCCTTGGCAGNNNNNNNNTAAAATAAGGCTAGTCCGTTATCAACTTGAAAAAGTGGCACCGAGTCGGTGCTTTTTT
4	Upper Stem	4^{11}	GTTTTANNNNN-ATACCAGCCGAAAGGCCCTTGGCAG--NNNNNNTAAAATAAGGCTAGTCCGTTATCAACTTGAAAAAGTGGCACCGAGTCGGTGCTTTTTT
5	Upper Stem	4^{12}	GTTTTANNNNN-ATACCAGCCGAAAGGCCCTTGGCAG-NNNNNNNTAAAATAAGGCTAGTCCGTTATCAACTTGAAAAAGTGGCACCGAGTCGGTGCTTTTTT
6	Upper Stem	4^{12}	GTTTTANNNNNNATACCAGCCGAAAGGCCCTTGGCAG--NNNNNNTAAAATAAGGCTAGTCCGTTATCAACTTGAAAAAGTGGCACCGAGTCGGTGCTTTTTT
7	Nexus	4^{4}	GTTTTAGAGCTA-----GAAA-----TAGCAAGTTAAAATAANN---ATACCAGCCGAAAGGCCCTTGGCAG---NNGTTATCAACTTGAAAAAGTGGCACCGAGTCGGTGCTTTTTTT
8	Nexus	4^{5}	GTTTTAGAGCTA-----GAAA-----TAGCAAGTTAAAATAANN---ATACCAGCCGAAAGGCCCTTGGCAG--NNNGTTATCAACTTGAAAAAGTGGCACCGAGTCGGTGCTTTTTTT
9	Nexus	4^{6}	GTTTTAGAGCTA-----GAAA-----TAGCAAGTTAAAATAANN---ATACCAGCCGAAAGGCCCTTGGCAG-NNNNGTTATCAACTTGAAAAAGTGGCACCGAGTCGGTGCTTTTTTT
10	Nexus	4^{7}	GTTTTAGAGCTA-----GAAA-----TAGCAAGTTAAAATAANN---ATACCAGCCGAAAGGCCCTTGGCAGNNNNNGTTATCAACTTGAAAAAGTGGCACCGAGTCGGTGCTTTTTTT
11	Nexus	4^{5}	GTTTTAGAGCTA-----GAAA-----TAGCAAGTTAAAATAANNN--ATACCAGCCGAAAGGCCCTTGGCAG---NNGTTATCAACTTGAAAAAGTGGCACCGAGTCGGTGCTTTTTTT
12	Nexus	4^{6}	GTTTTAGAGCTA-----GAAA-----TAGCAAGTTAAAATAANNN--ATACCAGCCGAAAGGCCCTTGGCAG--NNNGTTATCAACTTGAAAAAGTGGCACCGAGTCGGTGCTTTTTTT
13	Nexus	4^{7}	GTTTTAGAGCTA-----GAAA-----TAGCAAGTTAAAATAANNN--ATACCAGCCGAAAGGCCCTTGGCAG-NNNNGTTATCAACTTGAAAAAGTGGCACCGAGTCGGTGCTTTTTTT
14	Nexus	4^{8}	GTTTTAGAGCTA-----GAAA-----TAGCAAGTTAAAATAANNN--ATACCAGCCGAAAGGCCCTTGGCAGNNNNNGTTATCAACTTGAAAAAGTGGCACCGAGTCGGTGCTTTTTTT
15	Nexus	4^{6}	GTTTTAGAGCTA-----GAAA-----TAGCAAGTTAAAATAANNNN-ATACCAGCCGAAAGGCCCTTGGCAG---NNGTTATCAACTTGAAAAAGTGGCACCGAGTCGGTGCTTTTTTT
16	Nexus	4^{7}	GTTTTAGAGCTA-----GAAA-----TAGCAAGTTAAAATAANNNN-ATACCAGCCGAAAGGCCCTTGGCAG--NNNGTTATCAACTTGAAAAAGTGGCACCGAGTCGGTGCTTTTTTT
17	Nexus	4^{8}	GTTTTAGAGCTA-----GAAA-----TAGCAAGTTAAAATAANNNN-ATACCAGCCGAAAGGCCCTTGGCAG-NNNNGTTATCAACTTGAAAAAGTGGCACCGAGTCGGTGCTTTTTTT
18	Nexus	4^{9}	GTTTTAGAGCTA-----GAAA-----TAGCAAGTTAAAATAANNNN-ATACCAGCCGAAAGGCCCTTGGCAGNNNNNGTTATCAACTTGAAAAAGTGGCACCGAGTCGGTGCTTTTTTT
19	Nexus	4^{7}	GTTTTAGAGCTA-----GAAA-----TAGCAAGTTAAAATAANNNNNATACCAGCCGAAAGGCCCTTGGCAG---NNGTTATCAACTTGAAAAAGTGGCACCGAGTCGGTGCTTTTTTT
20	Nexus	4^{8}	GTTTTAGAGCTA-----GAAA-----TAGCAAGTTAAAATAANNNNNATACCAGCCGAAAGGCCCTTGGCAG--NNNGTTATCAACTTGAAAAAGTGGCACCGAGTCGGTGCTTTTTTT
21	Nexus	4^{9}	GTTTTAGAGCTA-----GAAA-----TAGCAAGTTAAAATAANNNNNATACCAGCCGAAAGGCCCTTGGCAG-NNNNGTTATCAACTTGAAAAAGTGGCACCGAGTCGGTGCTTTTTTT
22	Nexus	4^{10}	GTTTTAGAGCTA-----GAAA-----TAGCAAGTTAAAATAANNNNNATACCAGCCGAAAGGCCCTTGGCAGNNNNNGTTATCAACTTGAAAAAGTGGCACCGAGTCGGTGCTTTTTTT
23	Hairpin	4^{10}	GTTTTAGAGCTA-----GAAA-----TAGCAAGTTAAAATAAGGCTAGTCCGTTNN--NNNNATACCAGCCGAAAGGCCCTTGGCAG--NNNNGGCACCGAGTCGGTGCTTTTTT
24	Hairpin	4^{11}	GTTTTAGAGCTA-----GAAA-----TAGCAAGTTAAAATAAGGCTAGTCCGTTNN--NNNNATACCAGCCGAAAGGCCCTTGGCAG-NNNNNGGCACCGAGTCGGTGCTTTTTT
25	Hairpin	4^{12}	GTTTTAGAGCTA-----GAAA-----TAGCAAGTTAAAATAAGGCTAGTCCGTTNN--NNNNATACCAGCCGAAAGGCCCTTGGCAGNNNNNNGGCACCGAGTCGGTGCTTTTTT
26	Hairpin	4^{11}	GTTTTAGAGCTA-----GAAA-----TAGCAAGTTAAAATAAGGCTAGTCCGTTNNN-NNNNATACCAGCCGAAAGGCCCTTGGCAG--NNNNGGCACCGAGTCGGTGCTTTTTT
27	Hairpin	4^{12}	GTTTTAGAGCTA-----GAAA-----TAGCAAGTTAAAATAAGGCTAGTCCGTTNNN-NNNNATACCAGCCGAAAGGCCCTTGGCAG-NNNNNGGCACCGAGTCGGTGCTTTTTT
28	Hairpin	4^{12}	GTTTTAGAGCTA-----GAAA-----TAGCAAGTTAAAATAAGGCTAGTCCGTTNNNNNNNNATACCAGCCGAAAGGCCCTTGGCAG--NNNNGGCACCGAGTCGGTGCTTTTTT
29	Hairpin	4^{9}	GTTTCAGAGCTATGCTGGAAACAGCATAGCAAGTTGAAATAAGGNNNNNCCNN-ATNNGCCGATACCAGCCGAAAGGCCCTTGGCAG--CGACGGCACCGAGTCGGTGCTTTTTT
30	Hairpin	4^{10}	GTTTCAGAGCTATGCTGGAAACAGCATAGCAAGTTGAAATAAGGNNNNNCCNNNATNNGCCGATACCAGCCGAAAGGCCCTTGGCAG--CGACGGCACCGAGTCGGTGCTTTTTT
31	Hairpin	4^{10}	GTTTCAGAGCTATGCTGGAAACAGCATAGCAAGTTGAAATAAGGNNNN--TCCNNNNNN-GCCGTCGGGGTGCCCTTCTGCGTGAAGGCTGAGAAATACCCGTATCACCTGATCTGGATAATGCCAGCGTAGGGAACGACGGCACCGAGTCGGTGCTTTTTT
32	Hairpin	4^{11}	GTTTCAGAGCTATGCTGGAAACAGCATAGCAAGTTGAAATAAGGNNNN--TCCNNNNNNNGCCGTCGGGGTGCCCTTCTGCGTGAAGGCTGAGAAATACCCGTATCACCTGATCTGGATAATGCCAGCGTAGGGAACGACGGCACCGAGTCGGTGCTTTTTT
33	Hairpin	4^{11}	GTTTCAGAGCTATGCTGGAAACAGCATAGCAAGTTGAAATAAGGNNNNN-TCCNNNNNN-GCCGTCGGGGTGCCCTTCTGCGTGAAGGCTGAGAAATACCCGTATCACCTGATCTGGATAATGCCAGCGTAGGGAACGACGGCACCGAGTCGGTGCTTTTTT
34	Hairpin	4^{12}	GTTTCAGAGCTATGCTGGAAACAGCATAGCAAGTTGAAATAAGGNNNNN-TCCNNNNNNNGCCGTCGGGGTGCCCTTCTGCGTGAAGGCTGAGAAATACCCGTATCACCTGATCTGGATAATGCCAGCGTAGGGAACGACGGCACCGAGTCGGTGCTTTTTT
35	Hairpin	4^{12}	GTTTCAGAGCTATGCTGGAAACAGCATAGCAAGTTGAAATAAGGNNNNNNTCCNNNNNN-GCCGTCGGGGTGCCCTTCTGCGTGAAGGCTGAGAAATACCCGTATCACCTGATCTGGATAATGCCAGCGTAGGGAACGACGGCACCGAGTCGGTGCTTTTTT

Supplementary Table 2: Library sequences screened by FACS (see Methods). ligRNA^{+} was selected from library #29, while ligRNA^{-} was selected from library #22. Libraries #29 and #30 are based on the only clone isolated from library #26. Libraries #31–35 use the thiamine pyrophosphate aptamer[9]. Color coding of sgRNA domains is as in **Supplementary Table 1.** No functional ligRNAs were isolated from libraries #1-6 because the only initial hits isolated from these libraries were spacer dependent.

Supplementary Table 3

Name	Library	Picks	Ligand	Fold Change sgG1	sgG2	sgR1	sgR2	Sequence
	1–6	1/18	theo	10.2^{+}	—	1.0^{-}	—	GUUUUA--AAGGAUACCAGCCGAAAGGCCCUUGGCAGCUUUAGC-UAAAAUAAGGCUAGUCCGUUAUCAACUUGAAAAAGUGGCACCGAGUCGGUGCUUUUUU
	1–6	2/18	theo	12.4^{+}	—	1.2^{+}	—	GUUUUA-CCCGAAUACCAGCCGAAAGGCCCUUGGCAGUCUUCGC-UAAAAUAAGGCUAGUCCGUUAUCAACUUGAAAAAGUGGCACCGAGUCGGUGCUUUUUU
	1–6	1/18	theo	10.7^{+}	—	1.4^{+}	—	GUUUUA--AUCGAUACCAGCCGAAAGGCCCUUGGCAGCGGCUU--UAAAAUAAGGCUAGUCCGUUAUCAACUUGAAAAAGUGGCACCGAGUCGGUGCUUUUUU
	1–6	1/18	theo	11.3^{+}	—	1.1^{-}	—	GUUUUA--UAGGAUACCAGCCGAAAGGCCCUUGGCAGCUGCUCGCUAAAAUAAGGCUAGUCCGUUAUCAACUUGAAAAAGUGGCACCGAGUCGGUGCUUUUUU
	1–6	1/18	theo	9.0^{+}	—	1.1^{+}	—	GUUUUA-ACCUGAUACCAGCCGAAAGGCCCUUGGCAGCUGUAGA-UAAAAUAAGGCUAGUCCGUUAUCAACUUGAAAAAGUGGCACCGAGUCGGUGCUUUUUU
	1–6	1/18	theo	8.0^{+}	—	1.0^{+}	—	GUUUUA-CCGAGAUACCAGCCGAAAGGCCCUUGGCAGCCAGUCU-UAAAAUAAGGCUAGUCCGUUAUCAACUUGAAAAAGUGGCACCGAGUCGGUGCUUUUUU
	1–6	1/18	theo	7.5^{+}	—	1.4^{-}	—	GUUUUA--CCCGAUACCAGCCGAAAGGCCCUUGGCAGCGAGAGCUUAAAAUAAGGCUAGUCCGUUAUCAACUUGAAAAAGUGGCACCGAGUCGGUGCUUUUUU
	1–6	1/18	theo	8.2^{+}	—	1.3^{-}	—	GUUUUA-CGCGGAUACCAGCCGAAAGGCCCUUGGCAGCCUAGG--UAAAAUAAGGCUAGUCCGUUAUCAACUUGAAAAAGUGGCACCGAGUCGGUGCUUUUUU
	1–6	1/18	theo	7.7^{+}	—	1.0^{+}	—	GUUUUA-GCUGAAUACCAGCCGAAAGGCCCUUGGCAGUCGGGCU-UAAAAUAAGGCUAGUCCGUUAUCAACUUGAAAAAGUGGCACCGAGUCGGUGCUUUUUU
	1–6	1/18	theo	9.4^{+}	—	1.1^{-}	—	GUUUUA--CGUGAUACCAGCCGAAAGGCCCUUGGCAGCUUAUGC-UAAAAUAAGGCUAGUCCGUUAUCAACUUGAAAAAGUGGCACCGAGUCGGUGCUUUUUU
	1–6	1/18	theo	8.6^{+}	—	1.0^{+}	—	GUUUUA--UCGCAUACCAGCCGAAAGGCCCUUGGCAGGCCUGC--UAAAAUAAGGCUAGUCCGUUAUCAACUUGAAAAAGUGGCACCGAGUCGGUGCUUUUUU
	1–6	1/18	theo	6.0^{+}	—	1.2^{-}	—	GUUUUA--CCAGAUACCAGCCGAAAGGCCCUUGGCAGCUGCCAGCUAAAAUAAGGCUAGUCCGUUAUCAACUUGAAAAAGUGGCACCGAGUCGGUGCUUUUUU
	1–6	1/18	theo	6.4^{+}	—	1.3^{-}	—	GUUUUA--AUCGAUACCAGCCGAAAGGCCCUUGGCAGCGCUGCUUUAAAAUAAGGCUAGUCCGUUAUCAACUUGAAAAAGUGGCACCGAGUCGGUGCUUUUUU
	1–6	1/18	theo	33.6^{+}	—	1.3^{+}	—	GUUUUA--GCGGAUACCAGCCGAAAGGCCCUUGGCAGCCGGGCUUUAAAAUAAGGCUAGUCCGUUAUCAACUUGAAAAAGUGGCACCGAGUCGGUGCUUUUUU
	1–6	1/18	theo	17.9^{+}	—	1.2^{-}	—	GUUUUA--ACGGAUACCAGCCGAAAGGCCCUUGGCAGCCGGGCAUUAAAAUAAGGCUAGUCCGUUAUCAACUUGAAAAAGUGGCACCGAGUCGGUGCUUUUUU
	1–6	1/18	theo	8.0^{+}	—	1.2^{-}	—	GUUUUA--AUAGAUACCAGCCGAAAGGCCCUUGGCAGCAGCAGC-UAAAAUAAGGCUAGUCCGUUAUCAACUUGAAAAAGUGGCACCGAGUCGGUGCUUUUUU
	1–6	1/21	theo	6.6^{-}	—	—	—	GUUUUAAAGCUGAUACCAGCCGAAAGGCCCUUGGCAGCGGCGC--UAAAAUAAGGCUAGUCCGUUAUCAACUUGAAAAAGUGGCACCGAGUCGGUGCUUUUUU
	1–6	1/21	theo	6.0^{-}	—	—	—	GUUUUA-CCCUAAUACCAGCCGAAAGGCCCUUGGCAGUGGGGU--UAAAAUAAGGCUAGUCCGUUAUCAACUUGAAAAAGUGGCACCGAGUCGGUGCUUUUUU
	1–6	10/21	theo	6.9^{-}	—	1.0^{+}	—	GUUUUAGCGCUGAUACCAGCCGAAAGGCCCUUGGCAGCGUCGC--UAAAAUAAGGCUAGUCCGUUAUCAACUUGAAAAAGUGGCACCGAGUCGGUGCUUUUUU
	1–6	1/21	theo	3.9^{-}	—	—	—	GUUUUAGUCUGUAUACCAGCCGAAAGGCCCUUGGCAGAUAGAU--UAAAAUAAGGCUAGUCCGUUAUCAACUUGAAAAAGUGGCACCGAGUCGGUGCUUUUUU
	1–6	3/21	theo	3.7^{-}	—	—	—	GUUUUAACCUGCAUACCAGCCGAAAGGCCCUUGGCAGGUAGGU--UAAAAUAAGGCUAGUCCGUUAUCAACUUGAAAAAGUGGCACCGAGUCGGUGCUUUUUU
	1–6	2/21	theo	7.1^{-}	—	—	—	GUUUUA-ACCUCAUACCAGCCGAAAGGCCCUUGGCAGGUGGU---UAAAAUAAGGCUAGUCCGUUAUCAACUUGAAAAAGUGGCACCGAGUCGGUGCUUUUUU
	1–6	3/21	theo	5.6^{-}	—	—	—	GUUUUA-CCCCCAUACCAGCCGAAAGGCCCUUGGCAGGGGGGU--UAAAAUAAGGCUAGUCCGUUAUCAACUUGAAAAAGUGGCACCGAGUCGGUGCUUUUUU
	7–22	4/20	theo	8.6^{-}	—	—	—	GUUUUAGAGCUA-----GAAA-----UAGCAAGUUAAAAUAAGGGGAAUACCAGCCGAAAGGCCCUUGGCAGUCUCCGUUAUCAACUUGAAAAAGUGGCACCGAGUCGGUGCUUUUUU
*	7–22	5/20	theo	13.7^{-}	—	—	—	GUUUUAGAGCUA-----GAAA-----UAGCAAGUUAAAAUAAGUGGGAUACCAGCCGAAAGGCCCUUGGCAGCCUACGUUAUCAACUUGAAAAAGUGGCACCGAGUCGGUGCUUUUUU
ligRNA^{-}	—	—	theo	15.8^{-}	4.3^{-}	5.5^{-}	10.2^{-}	GUUUCAGAGCUAUGCUGGAAACAGCAUAGCAAGUUGAAAUAAGUGGGAUACCAGCCGAAAGGCCCUUGGCAGCCUACGUUAUCAACUUGAAAAAGUGGCACCGAGUCGGUGCUUUUUU
	7–22	4/20	theo	10.2^{-}	—	—	—	GUUUUAGAGCUA-----GAAA-----UAGCAAGUUAAAAUAAGAAGGAUACCAGCCGAAAGGCCCUUGGCAGCUUUCGUUAUCAACUUGAAAAAGUGGCACCGAGUCGGUGCUUUUUU
	7–22	1/20	theo	13.2^{-}	—	—	—	GUUUUAGAGCUA-----GAAA-----UAGCAAGUUAAAAUAAGGUG-AUACCAGCCGAAAGGCCCUUGGCAGCAUCUGUUAUCAACUUGAAAAAGUGGCACCGAGUCGGUGCUUUUUU
	7–22	1/20	theo	11.0^{-}	—	—	—	GUUUUAGAGCUA-----GAAA-----UAGCAAGUUAAAAUAAGGGGAAUACCAGCCGAAAGGCCCUUGGCAGUCUUCGUUAUCAACUUGAAAAAGUGGCACCGAGUCGGUGCUUUUUU
	7–22	1/20	theo	—	—	—	—	GUUUUAGAGCUA-----GAAA-----UAGCAAGUUAAAAUAAGGGGAAUACCAGCCGAAAGGCCCUUGGCAGUUUGCGUUAUCAACUUGAAAAAGUGGCACCGAGUCGGUGCUUUUUU
	7–22	2/20	theo	5.6^{-}	—	—	—	GUUUUAGAGCUA-----GAAA-----UAGCAAGUUAAAAUAAGCCCGAUACCAGCCGAAAGGCCCUUGGCAGCGAGUGUUAUCAACUUGAAAAAGUGGCACCGAGUCGGUGCUUUUUU
	7–22	1/20	theo	7.3^{-}	—	—	—	GUUUUAGAGCUA-----GAAA-----UAGCAAGUUAAAAUAAGGAAAUACCAGCCGAAAGGCCCUUGGCAG-UUCCGUUAUCAACUUGAAAAAGUGGCACCGAGUCGGUGCUUUUUU
	7–22	1/20	theo	—	—	—	—	GUUUUAGAGCUA-----GAAA-----UAGCAAGUUAAAAUAAGUG--AUACCAGCCGAAAGGCCCUUGGCAG--CACGUUAUCAACUUGAAAAAGUGGCACCGAGUCGGUGCUUUUUU
	23–28	6/6	theo	14.1^{+}	—	1.4^{+}	—	GUUUUAGAGCUA-----GAAA-----UAGCAAGUUAAAAUAAGGCUAGUCCCUU-UUCGCCGAUACCAGCCGAAAGGCCCUUGGCAGCGACGGCACCGAGUCGGUGCUUUUUU
	29–30	1/15	theo	9.7^{+}	—	4.8^{+}	4.4^{+}	GUUUCAGAGCUAUGCUGGAAACAGCAUAGCAAGUUGAAAUAAGGCGGUCCCGUCAUCAGCCGAUACCAGCCGAAAGGCCCUUGGCAGCGACGGCACCGAGUCGGUGCUUUUUU
	29–30	1/15	theo	4.5^{+}	1.3^{+}	5.4^{+}	—	GUUUCAGAGCUAUGCUUGAAACAGCAUAGCAAGUUGAAAUAAGGACGAACCGUAAUCCGCCGAUACCAGCCGAAAGGCCCUUGGCAGCGACGGCACCGAGUCGGUGCUUUUUU
	29–30	1/15	theo	9.6^{+}	1.7^{+}	3.8^{+}	—	GUUUCAGAGCUUUGCUGGAAACAGCAUAGCAAGUUGAAAUAAGGUCUGACCAGA-UCCGCCGAUACCAGCCGAAAGGCCCUUGGCAGCGACGGCACCGAGUCGGUGCUUUUUU
	29–30	1/15	theo	7.3^{+}	1.3^{+}	9.8^{+}	4.5^{+}	GUUUCAGAGCUUUGCUGGAAACAGCAUAGCAAGUUGAAAUAAGGUGACACCGCA-UCCGCCGAUACCAGCCGAAAGGCCCUUGGCAGCGACGGCACCGAGUCGGUGCUUUUUU
	29–30	1/15	theo	10.1^{+}	1.3^{+}	2.5^{+}	6.5^{+}	GUUUCAGAGCUAUGCUGGAAACAGCAUAGCAAGUUGAAAUAAGGUGGUACCAUA-UCCGCCGAUACCAGCCGAAAGGCCCUUGGCAGCGACGGCACCGAGUCGGUGCUUUUUU
	29–30	1/15	theo	7.5^{+}	1.4^{-}	7.6^{+}	6.1^{+}	GUUUCAGAGCUAUGCUGGAAACAGCAUAGCAAGUUGAAAUAAGGUAAACCCCUCAUCAGCCGAUACCAGCCGAAAGGCCCUUGGCAGCGACGGCACCGAGUCGGUGCUUUUUU
	29–30	2/15	theo	10.8^{+}	—	3.4^{+}	—	GUUUCAGAGCUAUGCUGGAAACAGAAUAGCAAGUUGAAAUAAGGAUCCUCCCGCAUGCGCCGAUACCAGCCGAAAGGCCCUUGGCAGCGACGGCACCGAGUCGGUGCUUUUUU
	29–30	1/15	theo	10.8^{+}	1.1^{-}	5.0^{+}	6.8^{+}	GUUUCAGAGCUAUGCUGGAAACAGCAUAGCAAGUUGAAAUAAGGACGUCCCGCA-UUCGCCGAUACCAGCCGAAAGGCCCUUGGCAGCGACGGCACCGAGUCGGUGCUUUUUU
	29–30	1/15	theo	7.7^{+}	—	4.8^{+}	—	GUUUCAGAGCUAUGCUGGAAACAGUAUAGCAAGUUGAAAUAAGGUUCGUCCGCCAUUCGCCGAUACCAGCCGAAAGGCCCUUGGCAGCGACGGCACCGAGUCGGUGCUUUUUU
ligRNA^{+}	29–30	1/15	theo	10.3^{+}	1.4^{-}	8.6^{+}	7.8^{+}	GUUUCAGAGCUAUGCUGGAAACAGCAUAGCAAGUUGAAAUAAGGGUGUCCCGUA-UACGCCGAUACCAGCCGAAAGGCCCUUGGCAGCGACGGCACCGAGUCGGUGCUUUUUU
	29–30	1/15	theo	7.2^{+}	—	3.8^{+}	3.8^{+}	GUUUCAGAGCUAUACUGGAAACAGCAUAGCAAGUUGAAAUAAGGGCACUCCUAA-UCCGCCGAUACCAGCCGAAAGGCCCUUGGCAGCGACGGCACCGAGUCGGUGCUUUUUU
ligRNA$^{+}_{2}$	29–30	1/15	theo	7.2^{+}	1.0^{-}	15.3^{+}	10.8^{+}	GUUUCAGAGC-AUGCUGGAAACAGCAUAGCAAGUUGAAAUAAGGUCUUCCCCGCAUCCGCCGAUACCAGCCGAAAGGCCCUUGGCAGCGACGGCACCGAGUCGGUGCUUUUUU
	29–30	1/15	theo	7.2^{+}	1.4^{-}	10.3^{+}	—	GUUUCAGAGCCAUGCUGGAAACAGCAUAGCAAGUUGAAAUAAGGACGGUCCCGCAUCCGCCGAUACCAGCCGAAAGGCCCUUGGCAGCGACGGCACCGAGUCGGUGCUUUUUU
	29–30	1/15	theo	11.2^{+}	1.2^{-}	3.2^{+}	—	GUUUCAGAGCUAUGCUGGAAACAGCAUAGCAAGUUGAAAUAAGGACUCUCCGUA-UCGGCCGAUACCAGCCGAAAGGCCCUUGGCAGCGACGGCACCGAGUCGGUGCUUUUUU
Fig S11: 8	31–35	2/20	tpp	—	—	4.3^{+}	—	GUUUCAGAGCUAUGCUGUAAACAGCAUAGCAAGUUGAAAUAAGGCGCAG-UCCGUCCCU-GCCGUCGGGGUGCCCUUCUGCGUGAAGGCUGAGAAAUACCCGUAUCACCUGAUCUGGAUAAUGCCAGCGUAGGGAACGACGGCACCGAGUCGGUGCUUUUUU
Fig S11: 30	31–35	1/20	tpp	—	—	5.3^{+}	—	GUUUCAGAGCUAUGCUGGAAACAGCAUAGCAAGUUGAAAUAAGGCCUG--UCCGUGAUCAGCCGUCGGGGUGCCCUUCUGCGUGAAGGCUGAGAAAUACCCGUAUCACCUGAUCUGGAUAAUGCCAGCGUAGGGAACGACGGCACCGAGUCGGUGCUUUUUU
Fig S11: 37	31–35	2/20	tpp	—	—	5.5^{+}	—	GUUUCAGAGCUAUGCUGGAAACAGCAUAGCAAGUUGAAAUAAGGGCAGG-UCCGUUCCCAGCCGUCGGGGUGCCCUUCUGCGUGAAGGCUGAGAAAUACCCGUAUCACCUGAUCUGGAUAAUGCCAGCGUAGGGAACGACGGCACCGAGUCGGUGCUUUUUU
Fig S11: 49	31–35	1/20	tpp	—	—	5.2^{+}	—	GUUUCAGAGCUAUGCUGGAAACAGCAUAGCAAGUUGAAAUAAGGCUAGG-UCCGUUCCCGGCCGUCGGGGUGCCCUUCUGCGUGAAGGCUGAGAAAUACCCGUAUCACCUGAUCUGGAUAAUGCCAGCGUAGGGAACGACGGCACCGAGUCGGUGCUUUUUU
Fig S11: 66	31–35	1/20	tpp	—	—	4.5^{+}	—	GUUUCAGAGCUUUGCUGGAAACAGCAUAGCAAGUUGAAAUAAGGCAGAGGUCCGUUCCU-GCCGUCGGGGUGCCCUUCUGCGUGAAGGCUGAGAAAUACCCGUAUCACCUGAUCUGGAUAAUGCCAGCGUAGGGAACGACGGCACCGAGUCGGUGCUUUUUU
Fig S11: 71	31–35	1/20	tpp	—	—	4.1^{+}	—	GUUUCAGAGCUAUGCUGGAAACAGCAUAGCAAGUUGAAAUAAGGUUCAC-UCCGUUCCCAGCCGUCGGGGUGCCCUUCUGCGUGAAGGCUGAGAAAUACCCGUAUCACCUGAUCUGGAUAAUGCCAGCGUAGGGAACGACGGCACCGAGUCGGUGCUUUUUU
Fig S11: 84	31–35	2/20	tpp	—	—	4.9^{+}	—	GUUUCAGAGCUAUGCUGGAAACAGCAUAGCAAGUUGAAAUAAGGGCGAG-UCCGUUCCCAGCCGUCGGGGUGCCCUUCUGCGUGAAGGCUGAGAAAUACCCGUAUCACCUGAUCUGGAUAAUGCCAGCGUAGGGAACGACGGCACCGAGUCGGUGCUUUUUU
Fig S11: 132	31–35	5/20	tpp	—	—	5.1^{+}	—	GUUUCAGAGCUAUGCUUGAAACAGCAUAGCAAGUUGAAAUAAGGCGCCAGUCCGUUCCU-GCCGUCGGGGUGCCCUUCUGCGUGAAGGCUGAGAAAUACCCGUAUCACCUGAUCUGGAUAAUGCCAGCGUAGGGAACGACGGCACCGAGUCGGUGCUUUUUU
Fig S11: 135	31–35	1/20	tpp	—	—	4.5^{+}	—	GUUUCAGAGCU-UGCUGGAAACAGCAUAGCAAGUUGAAAUAAGGUUUGA-UCCGUUCAGGGCCGUCGGGGUGCCCUUCUGCGUGAAGGCUGAGAAAUACCCGUAUCACCUGAUCUGGAUAAUGCCAGCGUAGGGAACGACGGCACCGAGUCGGUGCUUUUUU
Fig S11: 160	31–35	3/20	tpp	—	—	5.7^{+}	—	GUUUCAGAGCUAUGCUGUAAACAGCAUAGCAAGUUGAAAUAAGGCCCUG-UCCGCGAUCAGCCGUCGGGGUGCCCUUCUGCGUGAAGGCUGAGAAAUACCCGUAUCACCUGAUCUGGAUAAUGCCAGCGUAGGGAACGACGGCACCGAGUCGGUGCUUUUUU
Fig S11: 174	31–35	1/20	tpp	—	—	4.6^{+}	—	GUUUCAGAGCUAUGCUUGAAACAGCAUAGCAAGUUGAAAUAAGGCUUG--UCCGCGCUG-GCCGUCGGGGUGCCCUUCUGCGUGAAGGCUGAGAAAUACCCGUAUCACCUGAUCUGGAUAAUGCCAGCGUAGGGAACGACGGCACCGAGUCGGUGCUUUUUU

Supplementary Table 3: Ligand-sensitive sgRNAs isolated from FACS screen. Name: Names of designs described in the main text. The asterisk (*) denotes the design that was the precursor to ligRNA^{-}; we created ligRNA^{-} by transferring the nexus stem from this precursor into the optimized sgRNA scaffold described in ref.[10]. Library: Libraries (**Supplementary Table 2**) from which each design was isolated. Picks: The number of times each design appeared (first number) out of the total number of colonies picked to sequence from each library (second number). Ligand: The small molecule that each sgRNA was designed to sense (theo: theophylline; tpp: thiamine pyrophosphate). Fold Change: The fold change in fluorescence in response to the addition of the indicated ligand, measured by flow cytometry. All data in this table are single-replicate. The subcolumns (sgG1, sgG2, sgR1, sgR2) indicate the spacer used for each measurement. Superscripts indicate whether CRISPRi was activated (+) or inhibited (−) by the addition of ligand. Dashes indicate spacers that were not tested. Note that the designs selected from libraries 1–6 appear to be spacer-dependent, i.e. they are functional with the sgG1 spacer that was used in the selection but not with the sgR1 spacer (which for these libraries was not used in the selection). We therefore did not pursue these designs further. In contrast, the designs from libraries 29–30 were selected using both the sgG1 and sgR1 spacers as described in **Figure 2c**, and in validation experiments also function with the sgR2 spacer (the sgG2 spacer is not functional with any of the designs). Sequence: The sequence of the isolated design, color coded as in **Supplementary Table 1.** Note that the randomized regions on either side of the aptamer often form based-paired stems. Also note that for the nexus insertions (libraries 7–22), U95 and G63 (in ligRNA^{-} numbering, alternatively the third position from the 3' end of the nexus and the position at the 5' end of the nexus) are conserved in 17 and 20 of the 20 of the isolated sequences, respectively. This observation corroborates the importance of these positions in our mutagenesis experiments (**Supplementary Figure 4**).

Supplementary Table 4

Name	Sequence
T7 promoter	TATAGTAATAATACGACTCACTATAG
AAVS spacer	GGGGCCACTAGGGACAGGAT
sgG1 spacer	CATCTAATTCAACAAGAATT
sgR1 spacer	AACTTTCAGTTTAGCGGTCT
sgG2 spacer	AGTAGTGCAAATAAATTTAA
sgR2 spacer	TGGAACCGTACTGGAACTGC
folA spacer	TCCACGATGAGGTAACCCCA
Theophylline (theo) aptamer	ATACCAGCCGAAAGGCCCTTGGCAG
3-Methylxanthine (3mx) aptamer	ATACCAGCCGAAAGGCCATTGGCAG
Thiamine pyrophosphate (tpp) aptamer	TCGGGGTGCCCTTCTGCGTGAAGGCTGAGAAATACCCGTATCACCTGATCTGGATAATGCCAGCGTAGGGAA
Positive control	GTTTCAGAGCTATGCTGGAAACAGCATAGCAAGTTGAAATAAGGCTAGTCCGTTATCAACTTGAAAAAGTGGCACCGAGTCGGTGCTTTTTT
Negative control (G63C, G64C)	GTTTCAGAGCTATGCTGGAAACAGCATAGCAAGTTGAAATAACCCTAGTCCGTTATCAACTTGAAAAAGTGGCACCGAGTCGGTGCTTTTTT
ligRNA^{+}	GTTTCAGAGCTATGCTGGAAACAGCATAGCAAGTTGAAATAAGGG-TGTCCCGTATACGCCGATACCAGCCGAAAGGCCCTTGGCAGCGACGGCACCGAGTCGGTGCTTTTTT
ligRNA$^{+}_{2}$	GTTTCAGAGC-ATGCTGGAAACAGCATAGCAAGTTGAAATAAGGTCTTCCCCGCATCCGCCGATACCAGCCGAAAGGCCCTTGGCAGCGACGGCACCGAGTCGGTGCTTTTTT
ligRNA$^{+}_{3}$	GTTTCAGAGCTATGCTGGAAACAGCATAGCAAGTTGAAATAAGGC-TGTGCCGTATACGCCGATACCAGCCGAAAGGCCCTTGGCAGCGACGGCACCGAGTCGGTGCTTTTTT
ligRNA$^{+}_{4}$	GTTTCAGAGCTATGCTGGAAACAGCATAGCAAGTTGAAATAAGCG-TGTCGCGTATACGCGGATACCAGCCGAAAGGCCCTTGGCAGCCACGGCACCGAGTCGGTGCTTTTTT
ligRNA^{-}	GTTTCAGAGCTATGCTGGAAACAGCATAGCAAGTTGAAATAAGTGGGATACCAGCCGAAAGGCCCTTGGCAGCCTACGTTATCAACTTGAAAAAGTGGCACCGAGTCGGTGCTTTTTT
ligRNA$^{-}_{2}$	GTTTCAGAGCTATGCTGGAAACAGCATAGCAAGTTGAAATAAGAGGGATACCAGCCGAAAGGCCCTTGGCAGCCTTCGTTATCAACTTGAAAAAGTGGCACCGAGTCGGTGCTTTTTT
ligRNA$^{-}_{3}$	GTTTCAGAGCTATGCTGGAAACAGCATAGCAAGTTGAAATAAGTGGAATACCAGCCGAAAGGCCCTTGGCAGTCTACGTTATCAACTTGAAAAAGTGGCACCGAGTCGGTGCTTTTTT

Supplementary Table 4: DNA sequences of main constructs.

Supplementary Table 5

#	Spacer (with context)	Score	pos (% cut)				neg (% cut)				ligRNA^{+} (% cut)				ligRNA^{-} (% cut)				
			apo	*holo*	Δ	σ	*apo*	*holo*	Δ	σ	*apo*	*holo*	Δ	σ	*apo*	*holo*	Δ	σ	N
1	gcagGGGAACTCAAGAGCGGAGGGtggtca	0.622	91	90	-1	1	1	2	1	0	20	57	37	6	89	15	-74	3	3
2	acgcGGGTCTTACCTTTAATAGAAggggtc	0.523	94	94	0	2	3	2	-1	1	36	88	52	10	94	30	-64	13	3
3	cccgGGGTTATGCCTACGATGACCgggaat	0.582	95	95	-0	1	3	1	-2	2	43	79	36	17	93	24	-69	17	3
4	atcaGGGTGTAAGGATCGGAGTGCaggctc	0.518	91	92	1	3	0	0	0	0	7	30	23	6	87	8	-79	9	3
5	ctgtGGGATAAGGAGTTCTCGTGTaggggc	0.568	—	—	–	–	0	1	0	0	47	81	34	22	95	30	-65	25	3
6	gcagGGGCTTGTATTGGTTCTGAGtggata	0.605	90	89	-1	1	2	1	-1	1	27	81	54	5	90	44	-47	22	3
7	gaagGGGAAAGAACTCATTATCGTtggtgg	0.587	97	97	-0	0	3	2	-2	1	52	77	26	9	97	41	-56	4	3
8	ctttGGGCGGGTTGAATAGTCGTTgggtga	0.505	91	92	1	4	0	0	-0	0	28	71	42	11	69	24	-45	10	3
9	ttgaGGGCATGACCTAAACTCTATcggcaa	0.582	94	93	-1	1	4	2	-2	2	2	16	14	3	90	15	-75	4	3
10	taccGGGTTGTGATTGGTAACAGAtggggg	0.563	96	95	-0	1	0	0	-0	1	44	66	21	3	87	26	-61	7	3
11	acaaGGGACCATATAGTAGAACATgggcca	0.702	93	93	-0	1	5	3	-2	2	28	59	31	10	93	47	-46	19	4
12	tctcGGGGTCATCAGTATGAGTAAagggac	0.548	83	85	2	3	3	1	-2	2	35	58	22	6	89	33	-56	9	4
13	cgctGGGGATAACAAATGACCATGcggtct	0.728	97	97	-0	0	1	1	-1	1	46	64	19	17	71	22	-49	14	3
14	acgaGGGAGCTCACTATTCCAGGTcggtaa	0.688	94	94	-0	0	0	0	0	0	8	32	25	14	34	3	-31	14	3
15	gagaGGGTCAGACGGGAAGCCGGAcggtat	0.627	89	89	0	2	0	0	0	0	2	15	14	3	71	25	-46	16	3
16	gtacGGGTTAACGGATCCAACACGaggacg	0.708	97	97	-0	0	0	0	0	0	4	15	11	1	45	2	-43	7	3
17	ttagGGGTGCCTTTCCCACGAGCTgggtag	0.515	96	95	-2	2	0	0	0	0	5	24	19	1	1	3	2	0	3
18	ggccGGGCAGGGGCGTGAGAATCGtgggaa	0.522	90	90	1	1	0	0	-0	0	7	23	16	10	39	19	-20	16	3
19	acatGGGGGTATTCCGACTTGACAtggtgg	0.594	91	90	-0	0	0	0	-0	0	26	37	12	6	46	20	-26	7	3
20	gcagGGGAGTGGGACAGGAGTACGtgggaa	0.525	92	90	-2	0	0	0	0	0	3	9	6	3	46	9	-37	14	3
21	tatcGGGTTGGACTCTCTAACGATaggaaa	0.622	97	96	-1	1	0	0	0	0	12	18	6	4	50	8	-42	13	3
22	caccGGGCCAGCGTCGTGCAAGCGggggtg	0.510	94	91	-3	4	0	0	0	0	0	3	3	4	22	1	-21	12	3
23	gtgtGGGGTTAGAGGCTGAGTCCAgggtcc	0.534	95	94	-1	1	0	0	0	0	2	8	6	1	0	0	0	0	3
24	tcgaGGGCCCGGAGCCTGGCACGTtggaag	0.527	96	95	-1	1	0	0	0	0	0	0	0	0	0	0	0	0	2

Supplementary Table 5: Detailed results for the *in vitro* DNA cleavage assay in the context of different spacers (**Figure 2h** in the main text)**.** #: The spacer number in **Figure 2h**. Spacer (with context): In upper case is the 20 nt spacer sequence being tested. Note that for *in vitro* cleavage assays, sgRNAs were designed with 3 5' Gs for facile transcription by T7 polymerase. This would not represent a significant bottleneck for the sgRNA space in general as sgRNAs could also be designed with fewer 5' Gs, or synthesized chemically without requirement for 5' Gs. Moreover, the positions at the 5' end of the spacer are the most tolerant to mismatches[11]. In upper and lower case is the corresponding sequence present in the target DNA. This includes 10 bp of context and (to the right of the spacer) the NGG protospacer adjacent motif (PAM). These sequences were chosen as described in the Methods section. Score: The "Rule Set 2" score of the target sequence by the method of ref.[12], which predicts Cas9 cleavage efficiency for the target sequence. We only tested sequences with scores greater than 0.5 (approximately half of those generated). pos, neg, ligRNA^{+}, ligRNA^{-}: Cleavage data for the controls and indicated sgRNA. The *apo*, *holo*, Δ, σ, N columns are as in **Supplementary Table 1**.

Supplementary Table 6

Target	Forward	Reverse	Amplicon	Fig S14
16S rRNA	CTCTTGCCATCGGATGTGCCCA	CCAGTGTGGCTGGTCATCCTCTCA	105	✓
sfGFP	GTTCCGTGGCCAACACTTGTCACT	TACATAACCTTCGGGCATGGCACT	117	✓
pos,neg,ligRNA^{-}	CAACAAGAATTGTTTCAGAG	CGGTGCCACTTTTTCAAGTT	114	
	CAACAAGAATTGTTTCAGAG	AAAAGCACCGACTCGGTGCC	127	
	GAATTGTTTCAGAGCTATGC	CGGTGCCACTTTTTCAAGTT	108	✓
	GAATTGTTTCAGAGCTATGC	AAAAGCACCGACTCGGTGCC	121	
	GAGCTATGCTGGAAACAGCA	CGGTGCCACTTTTTCAAGTT	97	
	GAGCTATGCTGGAAACAGCA	AAAAGCACCGACTCGGTGCC	110	
ligRNA^{+}	CAACAAGAATTGTTTCAGAG	CGGGACACCCTTATTTCAAC	63	
	CAACAAGAATTGTTTCAGAG	GGTATCGGCGTATACGGGAC	77	
	CAACAAGAATTGTTTCAGAG	TTTCGGCTGGTATCGGCGTA	85	
	GAATTGTTTCAGAGCTATGC	CGGGACACCCTTATTTCAAC	57	
	GAATTGTTTCAGAGCTATGC	GGTATCGGCGTATACGGGAC	71	
	GAATTGTTTCAGAGCTATGC	TTTCGGCTGGTATCGGCGTA	79	✓
	GAGCTATGCTGGAAACAGCA	CGGGACACCCTTATTTCAAC	46	
	GAGCTATGCTGGAAACAGCA	GGTATCGGCGTATACGGGAC	60	
	GAGCTATGCTGGAAACAGCA	TTTCGGCTGGTATCGGCGTA	68	

Supplementary Table 6: qPCR primers. Target: The gene reported by the primer pair. Forward, Reverse: The sequences of the forward and reverse primers, respectively. Amplicon: The length of the amplicon in base pairs (bp). For each target, the pair of primers that gave the best amplification was chosen to validate for qPCR. Validation data for the primer pairs is shown in **Supplementary Figure 14**.

Supplementary Figure 1

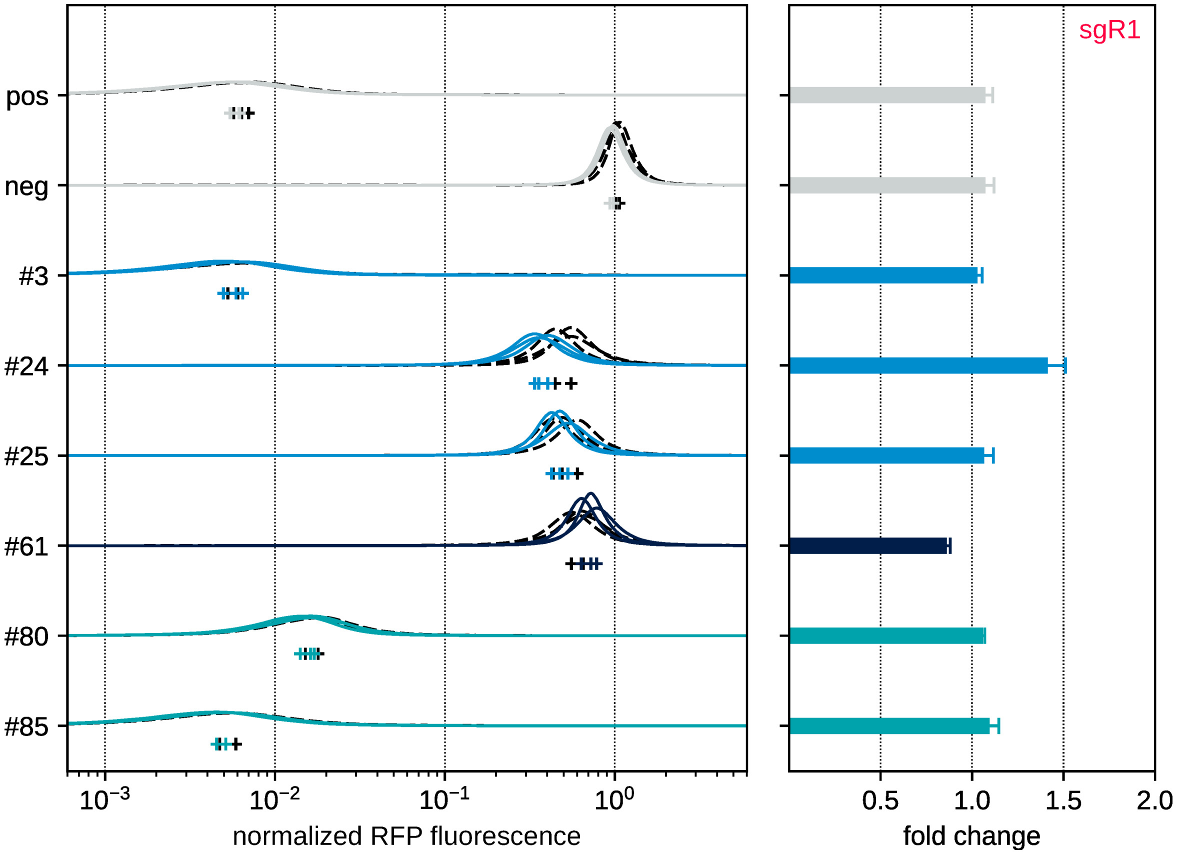

Supplementary Figure 1: Test of rational designs in *E. coli.* The strongest rational designs (determined by the *in vitro* cleavage assay, **Supplementary Table 1**) have only weak ligand-sensitivity in a CRISPRi-based assay in *E. coli*. Flow cytometry traces and fold changes for the rational designs that were tested in *E. coli*. The labels on the y-axis refer to **Supplementary Table 1**. Traces are color-coded by aptamer insertion site (defined in **Figure 1b**). RFP fluorescence values for each cell are normalized by both GFP fluorescence for that cell and the modes of the un-repressed control populations (i.e. *apo* and *holo*) measured for that replicate. All other lines and symbols in the left panel are as described in **Figure 2**. The bars in the right panel give the average fold change (specifically, apo/holo calculated using the modes of the normalized populations) of the replicates shown on the left. Error bars give the standard deviation. Data are from three experiments performed on the same day (for all other experiments reported in this paper, biological replicates were performed on different days). Designs #24 and #61 display a small ligand-dependency. For design #24 adding theophylline shifts the fluorescence distribution to the left (less fluorescence, indicating stabilization of the functional sgRNA conformation with ligand), whereas for design #61 the opposite is the case (shift to more fluorescence indicating destabilization of the functional sgRNA conformation with ligand). These changes are in the same direction as observed for these designs in the *in vitro* DNA cleavage assay (**Supplementary Table 1**), but much weaker.

Differences between *in vitro* and *in vivo* sgRNA activities have been reported in other systems[13]. One possible explanation is misfolding *in vivo*, which is alleviated *in vitro* because of an extra refolding step. This scenario could explain the case where constructs that were active *in vitro* were mostly inactive in *E. coli* irrespective of the presence of the ligand (#24, #25, #61). We also observe the case where a construct that is active and switchable *in vitro* is also active (represses) in *E. coli*, but unresponsive to ligand (#3, #80, #85). In this case, it is likely that the concentration of active ligRNA is above the sensitive range in both presence and absence of the ligand.

Supplementary Figure 2

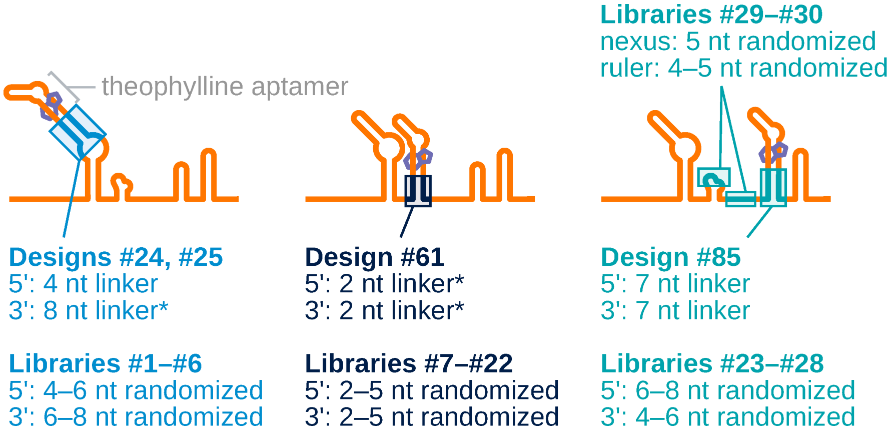

Supplementary Figure 2: Similarities between the rational designs and the FACS libraries. For each aptamer insertion site, the rational designs from **Figure 1d** (see also **Supplementary Table 1**) are compared to the libraries listed in **Supplementary Table 2**. The linker is defined as the sequence between the aptamer and the nearest region of the sgRNA scaffold shared between the designs and the libraries. Asterisks (*) indicate linkers in which not all nucleotides were mutated relative to the sgRNA scaffold.

Supplementary Figure 3

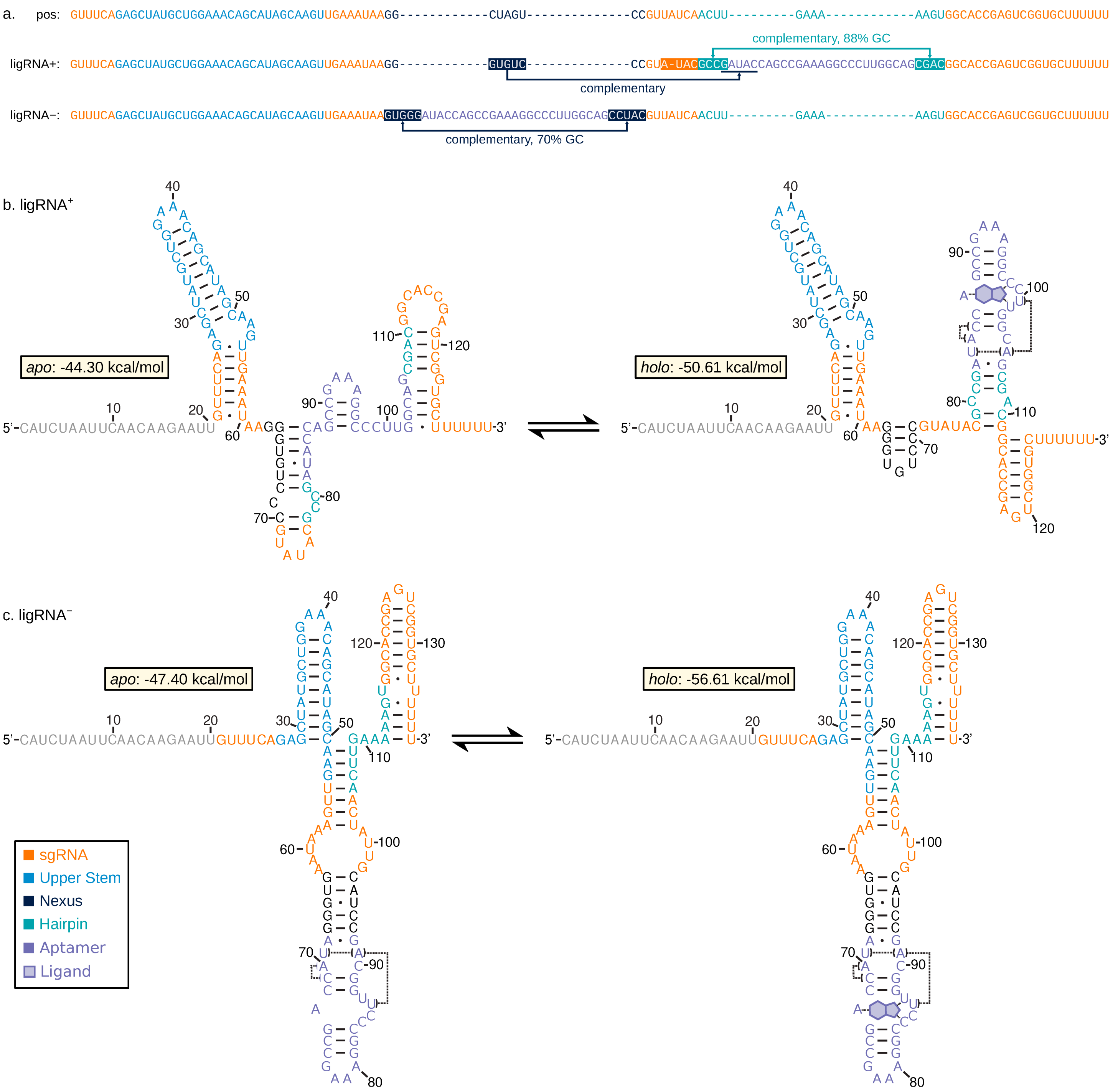

Supplementary Figure 3: Secondary structure predictions suggest mechanisms of ligand sensitivity. (**a**) Sequence alignment of the positive control sgRNA, ligRNA^{+}, and ligRNA^{-}. Nucleotides are color-coded by domain, and randomized positions are shaded. Several sequence features of the selected ligRNAs are noted. (**b,c**) Secondary structure and free energy predictions for ligRNA^{+} (**b**) and ligRNA^{-} (**b**) in both the apo and holo states, calculated as described in the Methods section. Nucleotides are color-coded by domain. (**b**) In the *apo* state of ligRNA^{+}, the nexus is predicted to base-pair with the aptamer, but in the *holo* state, the sgRNA is predicted to fold correctly. (**c**) The *apo* and *holo* states for ligRNA^{-} are predicted to have the same fold and neither prediction recapitulates the known sgRNA stems.

Supplementary Figure 4

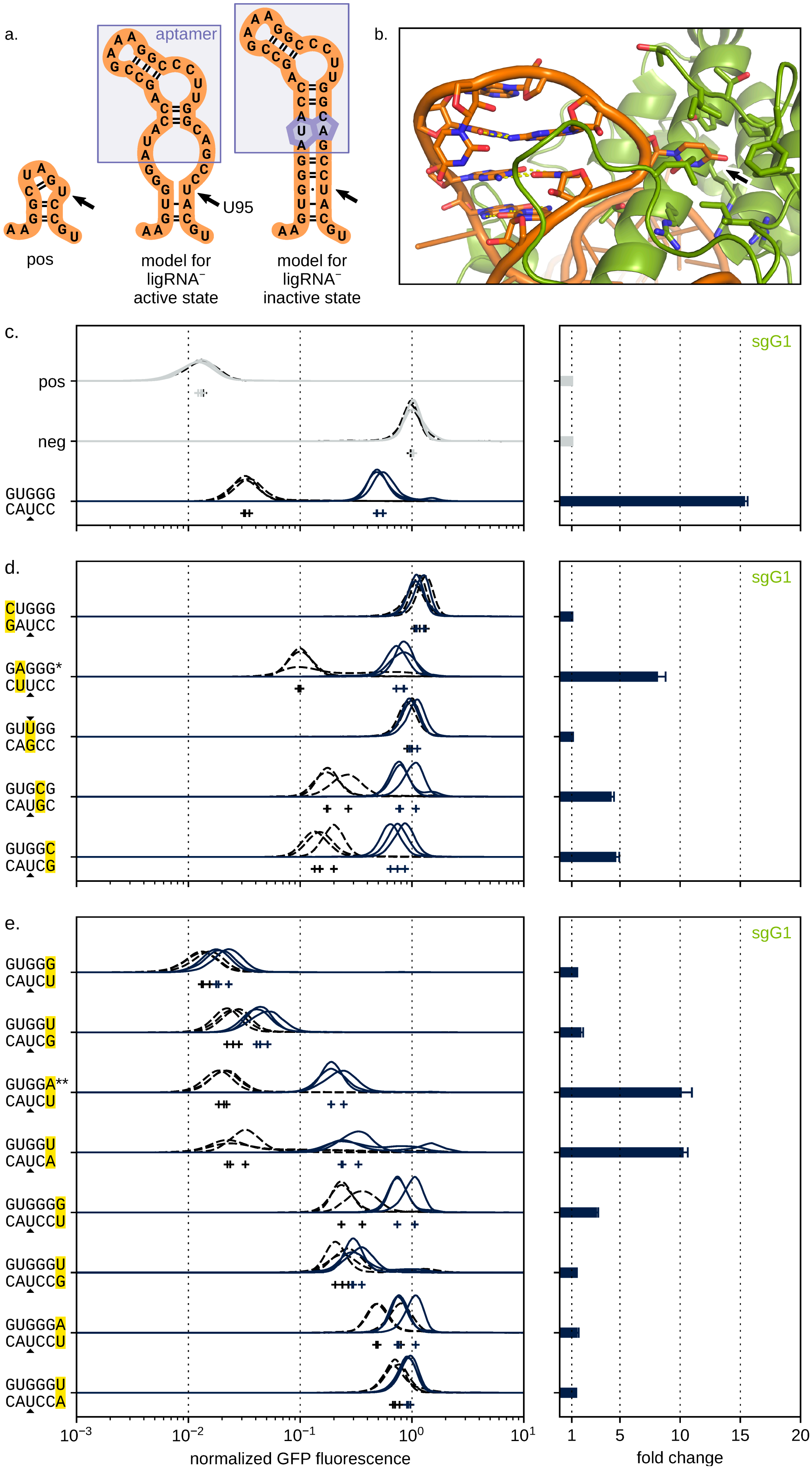

Supplementary Figure 4: Mechanistic insights into ligRNA$^-$ function. (**a**) Model of possible mechanism, where ligRNA$^-$ functions by sequestering the indicated uracil (U95) in the presence of the ligand. U95 is unpaired in wildtype sgRNA (left). Our hypothesis is that in ligRNA$^-$ U95 is unpaired to a larger extent in the *apo* state (center) than in the *holo* state (right). (**b**) A crystal structure of Cas9 in complex with an sgRNA[1] (PDB ID 4UN3) shows the indicated uracil flipped out (black arrow) and interacting with the Cas9 protein. (**c-e**) Flow cytometry traces (left) and fold changes (right) for different mutants of ligRNA$^-$. The labels show the sequence of the particular mutant being tested. Mutations relative to ligRNA$^-$ are highlighted in yellow. The uracil in question is indicated with a small triangle. The ligRNA$^-_2$ and ligRNA$^-_3$ variants (**Supplementary Table 4**; **Supplementary Figure 6**) are marked by * and **, respectively. GFP fluorescence values for each cell are normalized by both RFP fluorescence for that cell and the modes of the un-repressed control populations (i.e. *apo* and *holo*) measured for that replicate. There are three biological replicates for each mutant. Fold change bars that would have been less than one are inverted for clarity (i.e. holo/apo rather than apo/holo). All other lines and symbols are as described in **Supplementary Figure 2**. (**c**) Positive and negative controls, and ligRNA$^-$. (**d**) Strand-swap mutations for each position along the nexus stem. None of the mutants are as functional as ligRNA$^-$, but the design is tolerant to strand-swap mutation at positions 2, 4, and 5. As expected, position 3 containing the critical uracil is intolerant to mutation. The simple model in (**a**) does not explain the intolerance of position 1 however. (**e**) Modulating the strength of the base-pairs between the uracil and the aptamer has a predictable effect on function. From top to bottom, the mutants are arranged in the order of increasing base-pairing strength. Fluorescence distributions shifted to the left indicate stronger activation of the sgRNA (fluorescence is more effectively repressed). Our hypothesis (that ligRNA$^-$ works by sequestering the uracil upon ligand binding) predicts that weakening or strengthening the base-pairs between the uracil and the aptamer should increase or decrease activation, respectively. The clear downward diagonal trend in the populations supports this hypothesis and demonstrates that we can tune the dynamic range of ligRNA$^-$ to some extent. The third and fourth mutants (AU and UA) may be useful for applications where strong repression is desired because they show stronger repression that the original ligRNA$^-$ design in the absence of the ligand, although their dynamic range (10x) is somewhat narrower than that of ligRNA$^-$ (15x).

Supplementary Figure 5

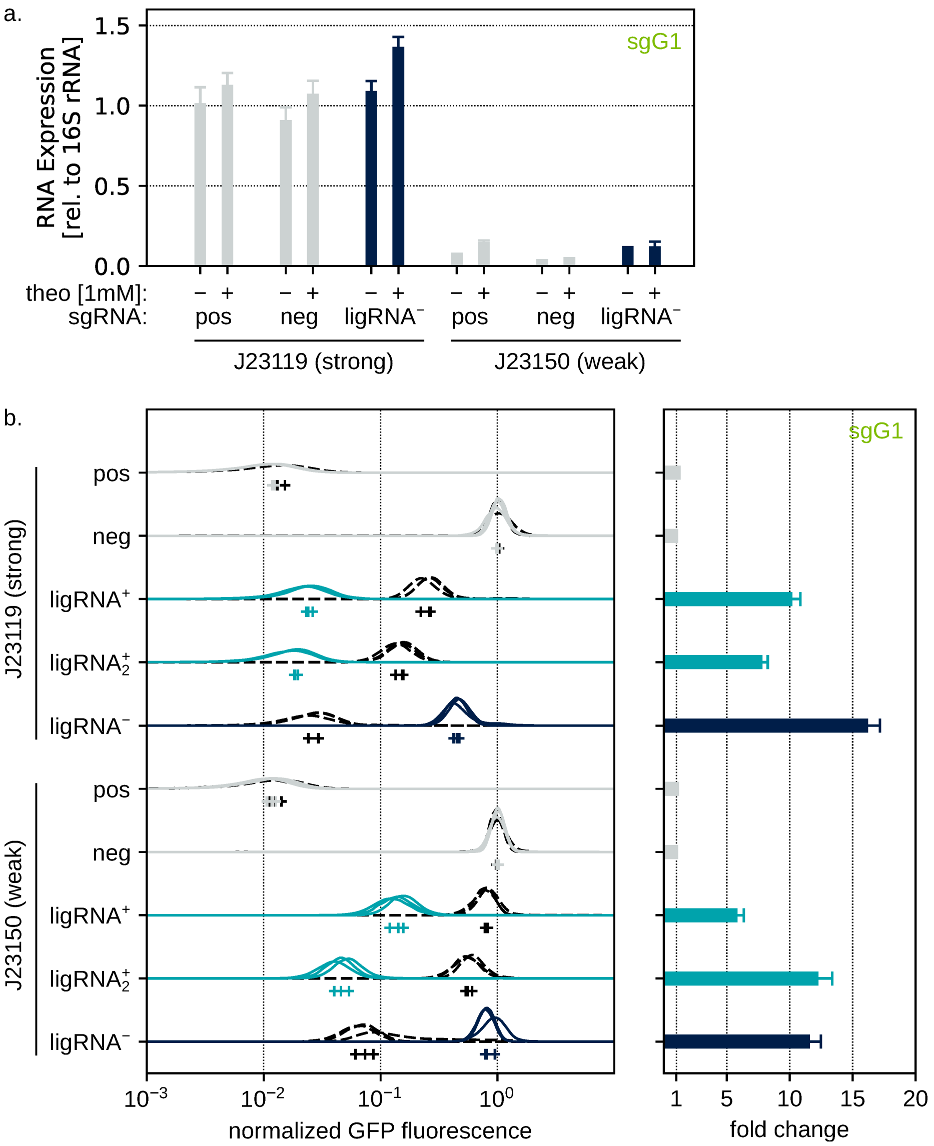

Supplementary Figure 5: ligRNA activity with two promoter strengths. (**a**) The strong (J23119) promoter results in significantly higher sgRNA expression than the weak (J23150) promoter, as expected. sgRNA levels were quantified using reverse transcription-quantitative polymerase chain reaction (RT-qPCR) as described in the Methods. The reported values are the fold change in sgRNA expression relative to the 16S rRNA. Primer validation data are shown in **Supplementary Figure 14**. No suitable primers were found for the ligRNA^{+} constructs, so data are only reported for the controls and ligRNA^{-}. (**b**) Flow cytometry traces (left) and fold changes (right) for three ligRNAs (ligRNA^{+} and ligRNA^{-} discussed in the main text and a third sequence termed ligRNA$^{+}_{2}$ isolated from the same screen as ligRNA^{+}, **Supplementary Table 3**) in the context of a strong (J23119) and a weak (J23150) constitutive promoter. GFP fluorescence values for each cell are normalized by both RFP fluorescence for that cell and the modes of the un-repressed control populations (i.e. *apo* and *holo*) measured for that replicate. There are three biological replicates for each ligRNA. Fold change bars that would have been less than one are inverted for clarity (i.e. holo/apo rather than apo/holo). All other lines and symbols are as described in **Supplementary Figure 1**. The alternate ligRNA$^{+}_{2}$ has a larger dynamic range with the weaker promoter, and may be useful for applications where lower concentrations of ligRNA are anticipated. Moreover, in the context of the weak promoter, the fluorescence distributions of ligRNA$^{+}_{2}$ in the absence of the ligand and the distributions of ligRNA^{-} in the presence of the ligand (inactive states) are close to the fluorescence distributions of the negative control, which may be useful when one of the desired states is full activation of gene expression.

Supplementary Figure 6

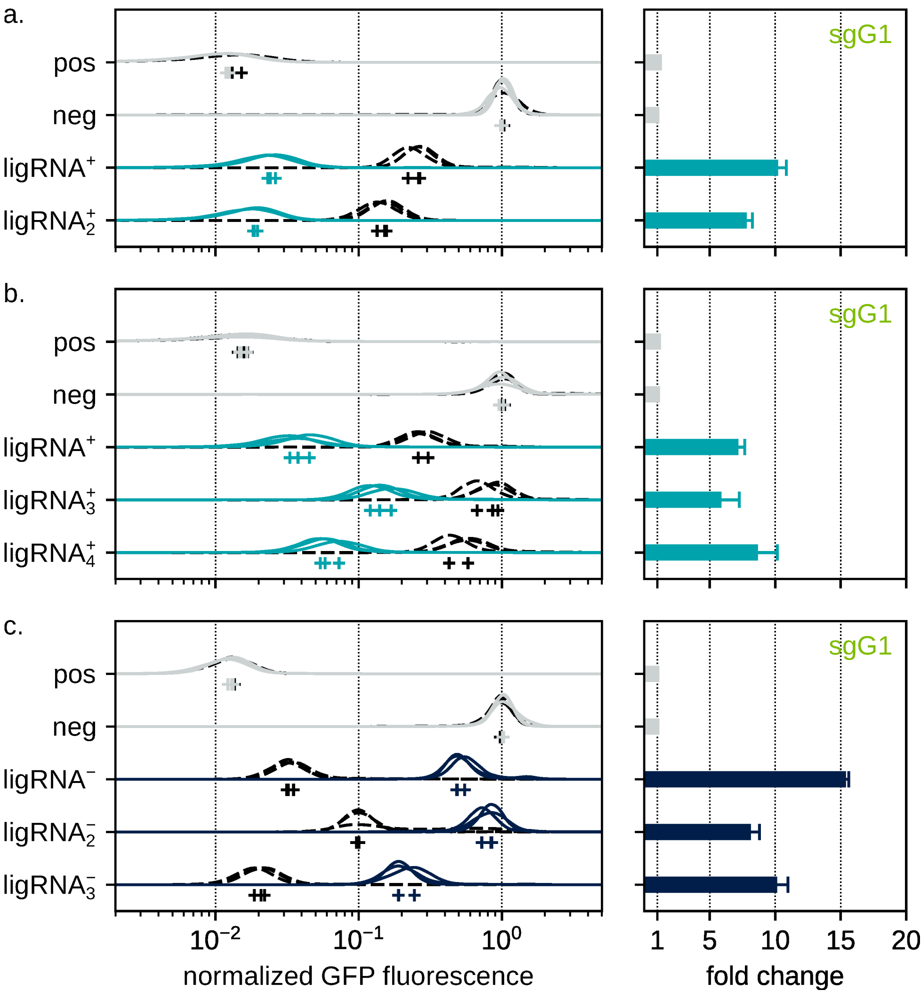

Supplementary Figure 6: ligRNA variants with shifted dynamic ranges. Flow cytometry traces (left) and fold changes (right) for sequence variants of ligRNA$^+$ (**a**,**b**) and ligRNA$^-$ (**c**) that maintain significant sensitivity to theophylline, but shift the dynamic range either towards maximum expression (ligRNA$^+_3$ and ligRNA$^+_4$ in the absence of the ligand, panel **b**; ligRNA$^-_2$ in the presence of the ligand, panel **c**) or maximum repression (ligRNA$^+_2$ in the presence of the ligand, panel **a**; ligRNA$^-_3$ in the absence of the ligand, panel **c**). See **Supplementary Table 4** for the sequences of these variants. GFP fluorescence values for each cell are normalized by both RFP fluorescence for that cell and the modes of the un-repressed control populations (i.e. *apo* and *holo*) measured for that replicate. There are three biological replicates for each mutant. Fold change bars that would have been less than one are inverted for clarity (i.e. holo/apo rather than apo/holo). All other lines and symbols are as described in **Supplementary 2**.

Supplementary Figure 7

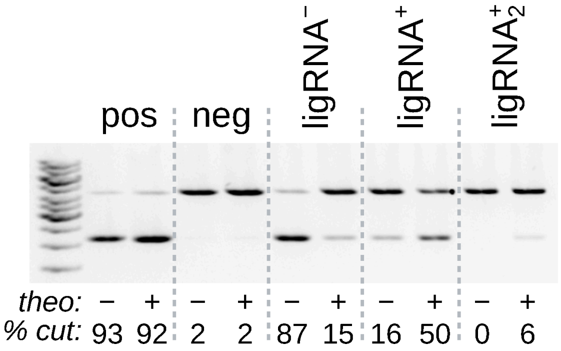

Supplementary Figure 7: Representative gel from the *in vitro* spacer assay. Shown is a single replicate for spacer #1 (**Figure 2h**). The upper and lower bands are uncleaved and cleaved DNA, respectively. Each design was tested in the absence and presence of theophylline. The amount of DNA cleavage was quantified by gel densiometry and is reported as a percentage below each lane.

Supplementary Figure 8

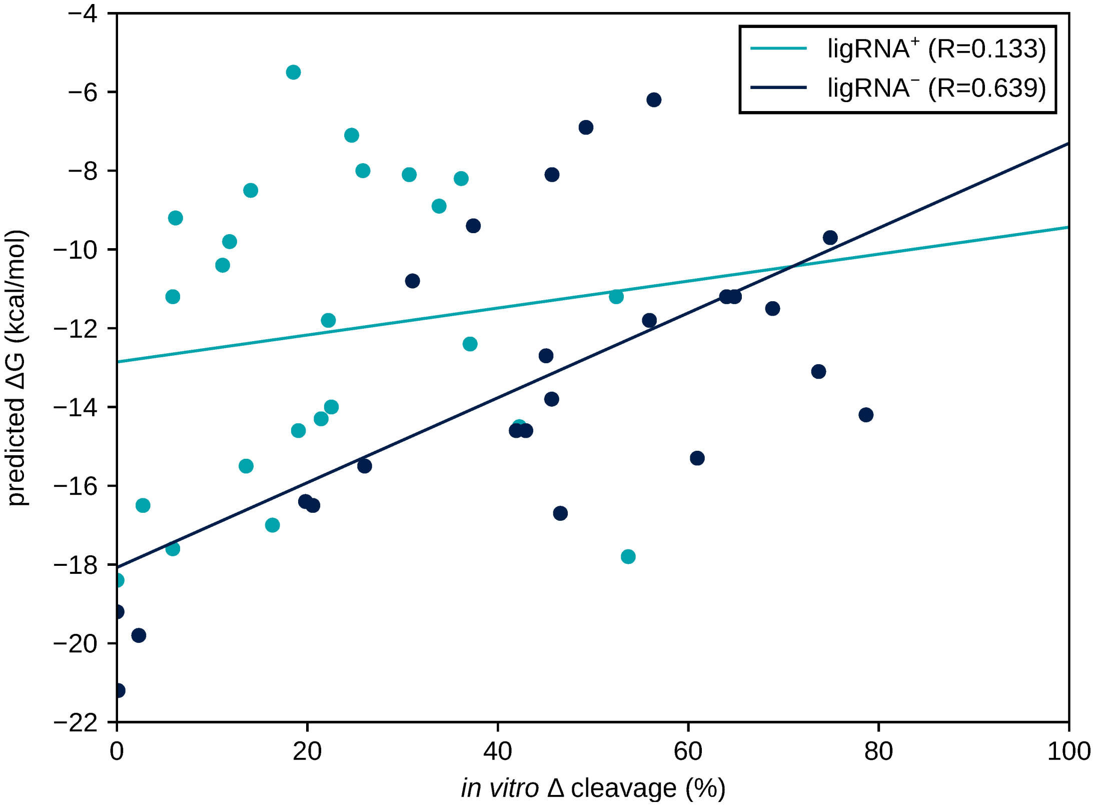

Supplementary Figure 8: Correlation between ligRNA function and the predicted binding free energy of base-pairing between the spacer and the aptamer insert. Binding free energies (y-axis) were calculated using the duplexfold method from the python3 API of the ViennaRNA package (version 2.4.3). This method returns the minimum binding energy between two strands of RNA considering only inter-strand base pairs. For each calculation, the first strand was one of the 24 20 nt spacers used in the *in vitro* spacer assay (**Supplementary Table 5**). The second strand was GCCGAUACCAGCCGAAAGGCCCUUGGCAGCGAC for ligRNA^{+} or GUGGGAUACCAGCCGAAAGGCCCUUGGCAGCCUAC for ligRNA^{-}. These sequences include both the aptamer and the randomized linker connecting the aptamer to the sgRNA scaffold. Percent cleavage values (x-axis) are the means of the replicates from the *in vitro* spacer assay (**Figure 2h, Supplementary Table 5**). Linear regressions (solid lines) and R-values are shown.

We note that a significant fraction of ligRNA^{+} constructs show low cleavage activities. Secondary structure prediction for our ligRNA^{+} constructs (**Supplementary Figure 3**) suggest that they function by sequestering the critical nexus region through base-pairing with the aptamer sequence in the ligand-free inactive state. Ligand binding to the aptamer is then predicted to relieve this sequestration and stabilize a functional sgRNA conformation. To allow for complementary base-pairing with the aptamer in the inactive state, the selection experiment yielded slightly altered nexus sequences. As a consequence, the ligRNA^{+} constructs might have overall intrinsically lower affinity for Cas9, because the nexus makes critical interactions with the Cas9 protein. Future design efforts could add constraints to keep the nexus sequence as native-like as possible.

Supplementary Figure 9

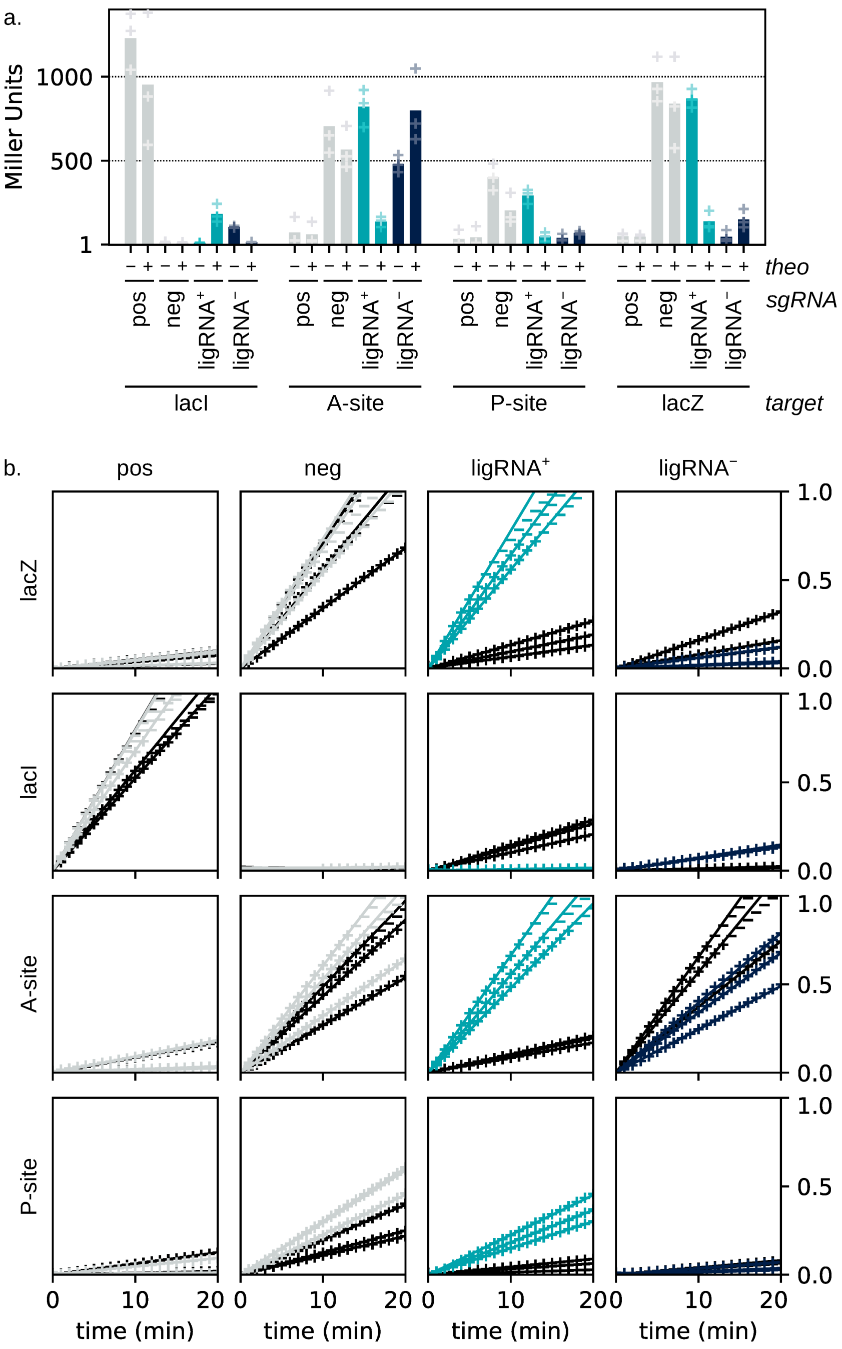

Supplementary Figure 9: ligRNA function targeting the *E. coli lac* operon. (**a**) β-galactosidase activities in Miller units for *E. coli* grown in the presence or absence of theophylline, expressing either a control sgRNA (pos or neg, grey), ligRNA^{+} (teal), or ligRNA^{-} (navy), with spacers targeting different components of the *lac* operon (lacI, A-site, P-site, lacZ). Note that, as expected, targeting lacI has the opposite effect on activity as targeting any of the other sites. Data are from three biological replicates; bar heights represent the mean and data for each replicate are shown as faded plus marks. (**b**) β-galactosidase activity data underlying the data above and fold changes reported in **Figure 3b**. Absorbance at 420 nm increases as β-galactosidase catalyzes the release of o-nitrophenol from o-nitrophenol-β-D-galactopyranoside (ONPG). Each column of plots is a different sgRNA, and each row is a different endogenous target. Linear fits to the initial rate of each reaction are shown as solid lines. Data points included in the fit are shown as "+" signs, while those excluded from the fit (due to the reaction slowing as ONPG becomes limiting) are shown as "−" signs. ΔA420: A baseline is subtracted from each trace to make the linear fits pass through the origin. Note that targeting *lacI* is expected to cause an increase in β-galactosidase activity, while targeting *lacZ* or the two transcriptional operator sites (A, P) is expected to cause a decrease.

Supplementary Figure 10

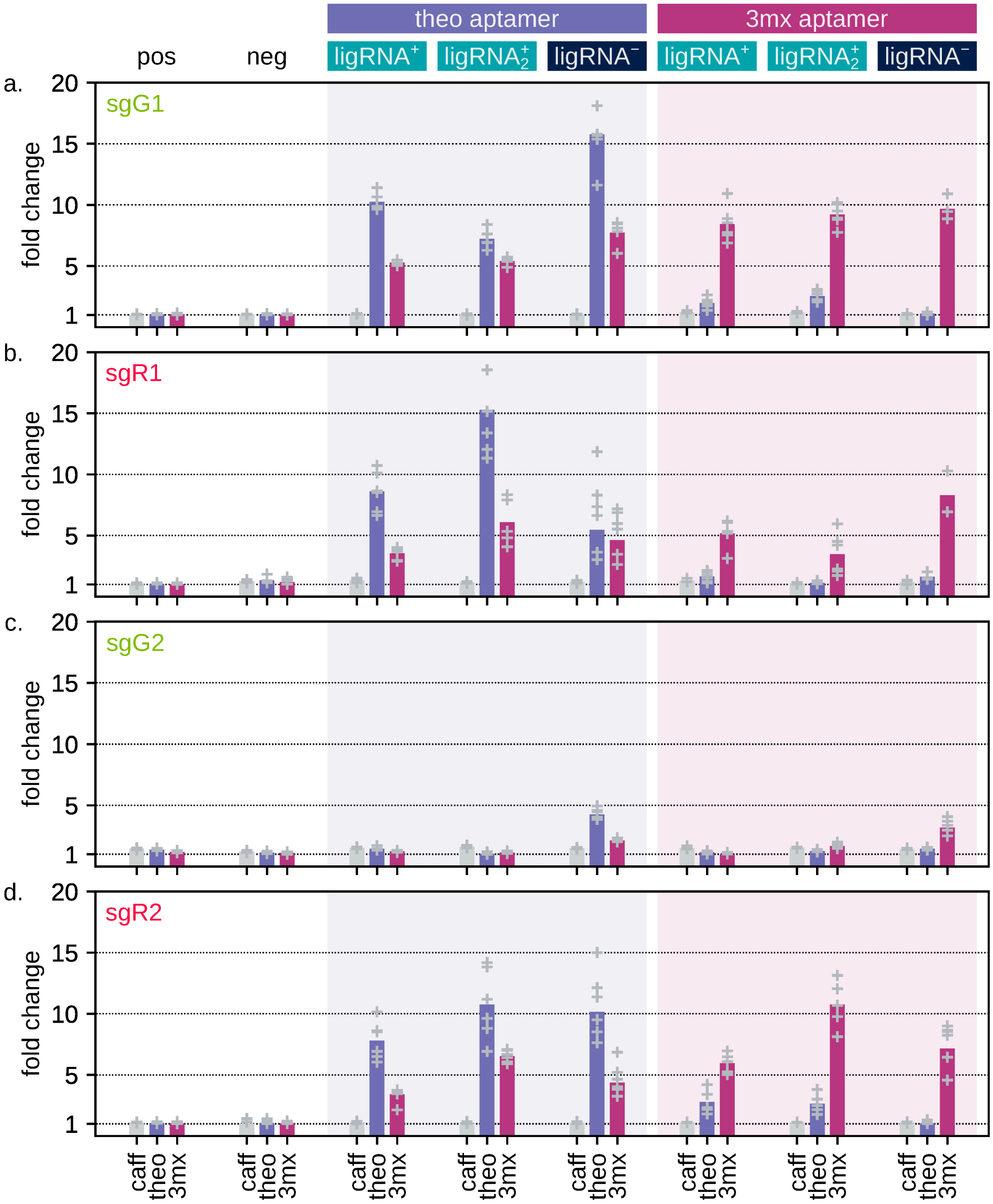

Supplementary Figure 10: ligRNA function with two different aptamers, four different spacers and three different ligands. We built versions of each ligRNA with both the theophylline aptamer (purple heading) and the 3-methylxanthine aptamer (magenta heading) and then tested them with the three different ligands indicated at the bottom: caffeine (caff, grey bars), theophylline (theo, purple bars), and 3-methylxanthine (3mx, magenta bars). Caffeine is a negative control; it is chemically similar to theophylline and 3-methylxanthine, but is not expected to bind to either aptamer at the concentrations used. The reported fold changes are relative to treatment with no ligand and are calculated from the modes of fluorescence distributions measured by flow cytometry. Bar heights represent the mean and data for each replicate are shown as grey plus marks. Panels (**a-d**) show data for the sgG1, sgR1, sgG2, and sgR2 spacers (**Supplementary Table 4**), respectively. Note that the theophylline aptamer (purple shading) is expected to be sensitive to both theophylline and 3-methylxanthine, but that the 3-methylxanthine aptamer (magenta shading) is expected to be specific to its ligand 3-methylxanthine with much reduced or no sensitivity to theophylline. The tested ligRNAs behave as expected with the different aptamers and ligands (note that none of the ligRNAs except ligRNA$^-$ showed significant ligand sensitivity with the sgG2 spacer, which we expected based on our prior results shown in **Supplementary Table 3**).

Supplementary Figure 11

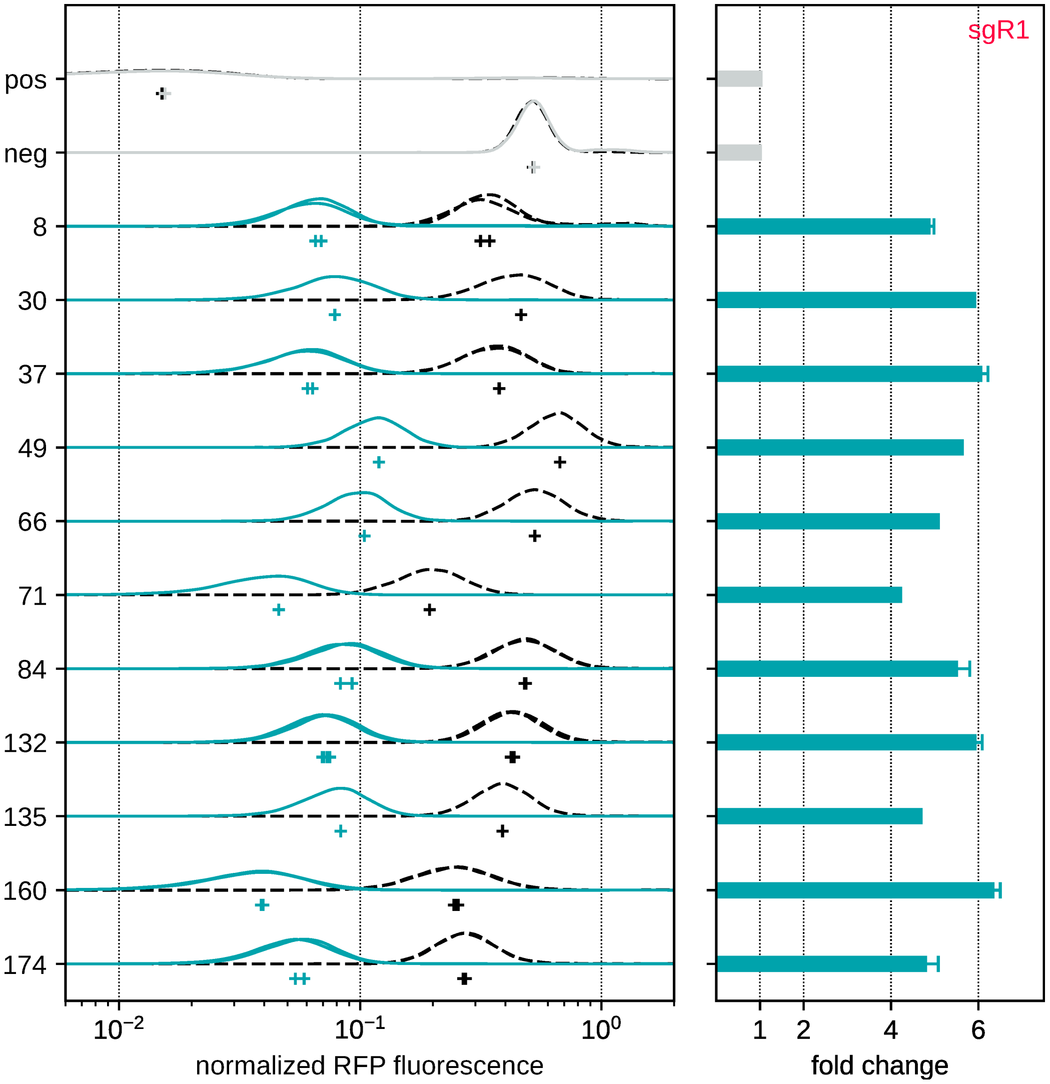

Supplementary Figure 11: Thiamine-sensitive ligRNAs. Flow cytometry traces (left) and fold changes (right) for the designs resulting from a screen for thiamine-sensitive ligRNAs. All lines and symbols are as described in **Supplementary Figure 1**, except that the solid lines refer to the presence of thiamine rather than theophylline. Fold-change in RFP repression in response to thiamine was measured for 188 of the colonies isolated from the screen. The 20 with the greatest fold change were sequenced, resulting in 11 unique designs (**Supplementary Table 3**). The y-axis label indicates the colony number of each design. Each colony was tested once, but some designs were present in multiple colonies and have 2–5 replicates as a result (for these cases, error bars give the standard deviation). Note that the sgR1 spacer was used in both the second stage of the screen (**Figure 2c**, screens #3 and 4) and the validation of individual colonies shown here.

Supplementary Figure 12

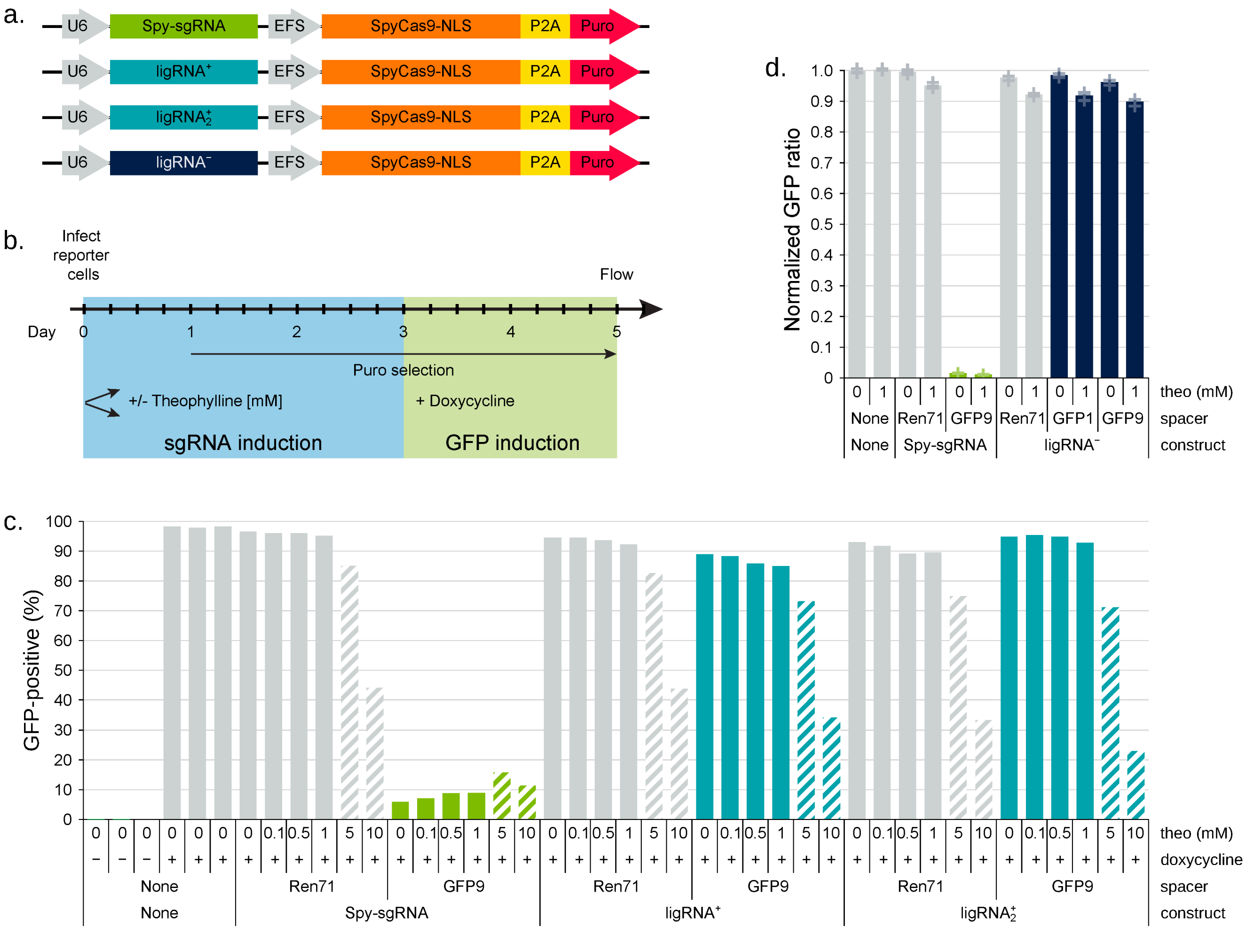

Supplementary Figure 12: **Test of ligRNA-mediated target editing in mammalian cell lines**. (**a**) Vector maps of lentiviral constructs expressing Cas9 and either a standard sgRNA as control, or a ligRNA. (**b**) Schematic of the GFP knockout assay in a HEK293T-based reporter cell line, referred to as HEK-RT1[14], with doxycycline-controlled GFP expression. (**c**) ligRNA^{+} does not exhibit theophylline-dependent editing in this assay. Theophylline concentrations from 0.1 to 10 mM were tested. Concentrations above 1 mM caused severe cell death (dashed bars). sgGFP9 (CCGGCAAGCTGCCCGTGCCC) targets the GFP of the reporter cell line. sgRen71 (TAGGAATTATAATGCTTATC) is a negative control sgRNA. Note, ligRNA^{+} resulted in a slight decrease of GFP-positive cells, but this trend began in absence of theophylline. (**d**) ligRNA^{-} does not exhibit theophylline-dependent editing in this assay. The slight differences between the 0 mM and 1 mM bars cannot be attributed to ligRNA^{-}, because the Ren71 control has a similar difference; in addition, we would expect editing with ligRNA^{-} to be inhibited, not activated, by theophylline. sgGFP1 (CCTCGAACTTCACCTCGGCG). Bar heights reflect the mean with individual measurements shown as faded plus marks.

Supplementary Figure 13

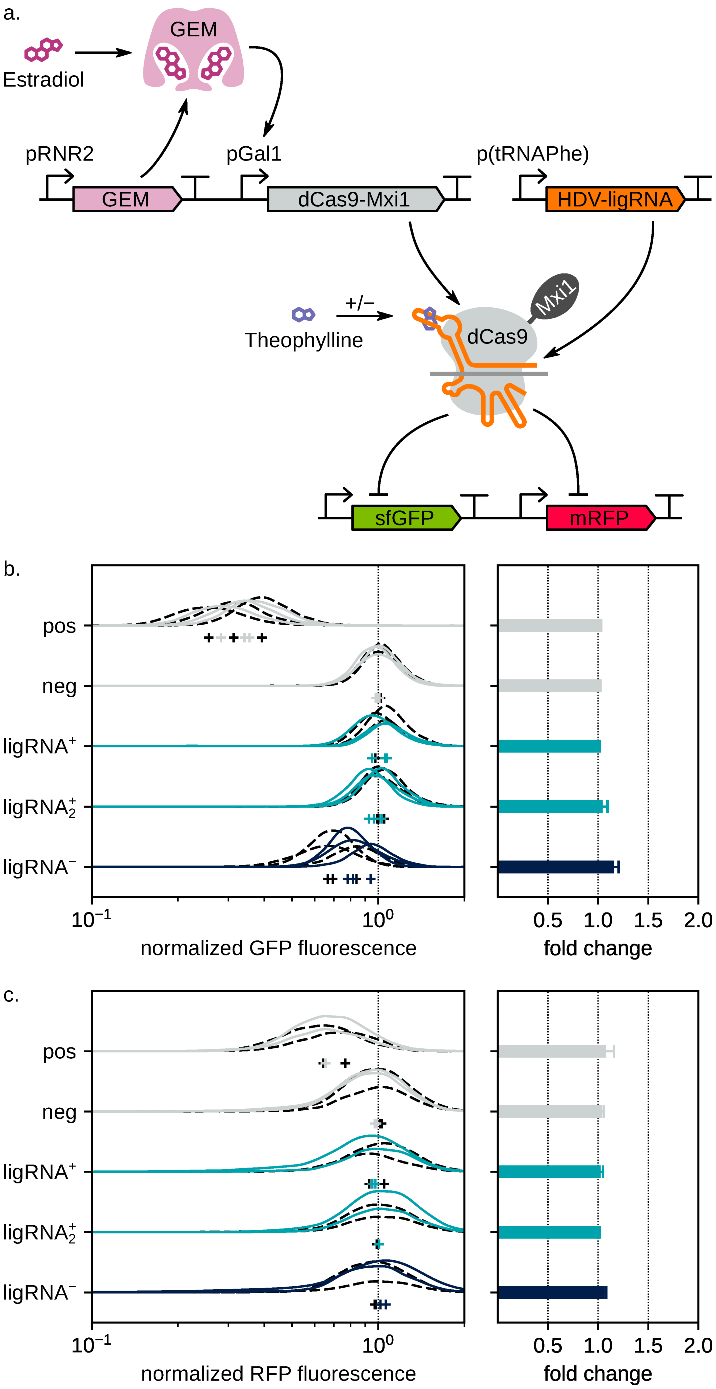

Supplementary Figure 13: **Test of ligRNA-mediated gene repression in yeast cells**. Flow cytometry traces (left) and fold changes (right) for the ligRNAs and positive and negative controls in a CRISPRi assay in *S. cerevisiae*. (**a**) Schematic depicting the various constructs in the engineered yeast strains. (**b**) GFP repression with the sgG2 spacer. ligRNA^{+} and ligRNA$^{+}_{2}$ do not exhibit ligand-dependent activity in this assay. ligRNA^{-} exhibits a weak effect in the expected direction. (**c**) RFP repression with the sgR2 spacer does not show ligand dependence in this assay. The theophylline concentration in the plus ligand condition was 2.5 mM. The expression of dCas9-Mxi1 was induced with 125nM estradiol. Fluorescence values for each cell are normalized by side scatter (SSC) and the modes of the un-repressed control populations (i.e. *apo* and *holo*) measured for that replicate. There are three biological replicates for each experiment. Fold change bars that would have been less than one are inverted for clarity (i.e. holo/apo rather than apo/holo). All other lines and symbols are as described in **Supplementary Figure 1**.

Supplementary Figure 14

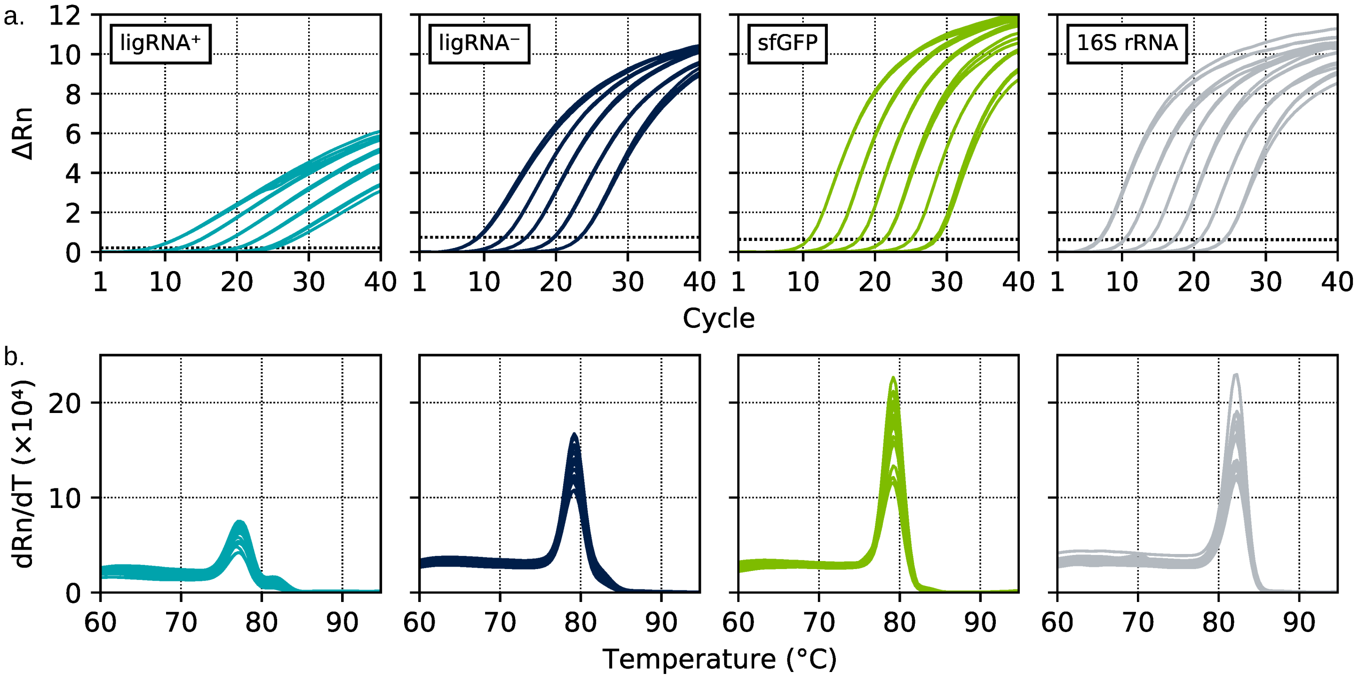

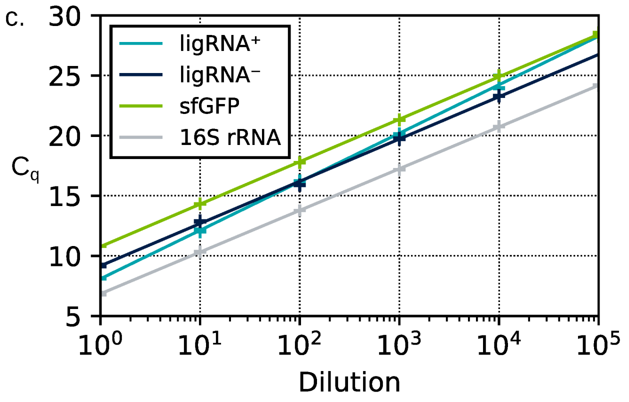

d.

Target	Slope	Intercept	R^2	Efficiency
ligRNA⁺	4.04	8.09	0.9981	~~76.9%~~
ligRNA⁻	3.52	9.17	0.9987	92.4%
sfGFP	3.53	10.76	0.9998	91.9%
16S rRNA	3.47	6.84	0.9999	94.2%

Supplementary Figure 14: qPCR primer validation. (**a**) PCR amplification curves for four different primer pairs (labeled "ligRNA⁺", "ligRNA⁻", "sfGFP", and "16S rRNA"). In each reaction, the template was reverse-transcribed total cellular RNA. The template for the ligRNA⁺ and ligRNA⁻ primer pairs came from strains expressing the corresponding ligRNAs (in the absence of theophylline). The template for the remaining primer pairs came from the strain expressing the negative control sgRNA (also in the absence of theophylline). For each primer pair, at least 5 10-fold serial dilutions of the template were measured. Three technical replicates were performed for each primer/dilution combination. ΔRn is the ratio of SYBR Green fluorescence (which should be proportional to the amount of double-stranded DNA) to ROX fluorescence (which should be independent of the amount of double-stranded DNA), shifted to a baseline value of 0. The C_q threshold is illustrated with a black dotted line. (**b**) Melting curves for the products of the above qPCR reactions. dRn/dT is the derivative of SYBR Green fluorescence (ΔRn) with respect to temperature. A single peak is consistent with all of the product DNA having the same sequence and the amplification being specific for the intended transcript. (**c**) The relationship between the dilution of the template DNA and the cycle at which ΔRn exceeds the C_T threshold. Data points are derived from the plots in panel (**a**) and are shown as plus marks. Linear fits are shown as solid lines. (**d**) Parameters of the linear fits from panel (**c**). R^2 is the coefficient of determination. We considered R^2>0.98 to be acceptable. Efficiency is the number of doublings per PCR cycle, expressed as a percent. We considered efficiencies between 90% and 110% to be acceptable. Values outside the acceptable range for either parameter are indicated with a strike-through. Due to their poor efficiency, the ligRNA⁺ primers were not used in any subsequent experiments.

Supplementary References

1. Nishimasu, H., Ran, F.A., Hsu, P.D., Konermann, S., Shehata, S.I., Dohmae, N., Ishitani, R., Zhang, F. & Nureki, O. Crystal structure of Cas9 in complex with guide RNA and target DNA. *Cell* **156**, 935-49 (2014).
2. Briner, A.E., Donohoue, P.D., Gomaa, A.A., Selle, K., Slorach, E.M., Nye, C.H., Haurwitz, R.E., Beisel, C.L., May, A.P. & Barrangou, R. Guide RNA functional modules direct Cas9 activity and orthogonality. *Mol Cell* **56**, 333-9 (2014).
3. Zimmermann, G.R., Jenison, R.D., Wick, C.L., Simorre, J.P. & Pardi, A. Interlocking structural motifs mediate molecular discrimination by a theophylline-binding RNA. *Nat Struct Biol* **4**, 644-9 (1997).
4. Jiang, H., Ling, K., Tao, X. & Zhang, Q. Theophylline detection in serum using a self-assembling RNA aptamer-based gold nanoparticle sensor. *Biosensors and Bioelectronics* **70**, 299-303 (2015).
5. Chen, A., Yan, M. & Yang, S. Split aptamers and their applications in sandwich aptasensors. *TrAC Trends in Analytical Chemistry* **80**, 581 - 593 (2016).
6. Lynch, S.A. & Gallivan, J.P. A flow cytometry-based screen for synthetic riboswitches. *Nucleic Acids Res* **37**, 184-92 (2009).
7. Muranaka, N., Abe, K. & Yokobayashi, Y. Mechanism-guided library design and dual genetic selection of synthetic OFF riboswitches. *Chembiochem* **10**, 2375-81 (2009).
8. Werstuck, G. & Green, M.R. Controlling gene expression in living cells through small molecule-RNA interactions. *Science* **282**, 296-8 (1998).
9. Wieland, M., Benz, A., Klauser, B. & Hartig, J.S. Artificial ribozyme switches containing natural riboswitch aptamer domains. *Angew Chem Int Ed Engl* **48**, 2715-8 (2009).
10. Dang, Y., Jia, G., Choi, J., Ma, H., Anaya, E., Ye, C., Shankar, P. & Wu, H. Optimizing sgRNA structure to improve CRISPR-Cas9 knockout efficiency. *Genome Biol* **16**, 280 (2015).
11. Fu, Y., Sander, J.D., Reyon, D., Cascio, V.M. & Joung, J.K. Improving CRISPR-Cas nuclease specificity using truncated guide RNAs. *Nat Biotechnol* **32**, 279-284 (2014).
12. Doench, J.G., Fusi, N., Sullender, M., Hegde, M., Vaimberg, E.W., Donovan, K.F., Smith, I., Tothova, Z., Wilen, C., Orchard, R., Virgin, H.W., Listgarten, J. & Root, D.E. Optimized sgRNA design to maximize activity and minimize off-target effects of CRISPR-Cas9. *Nat Biotechnol* **34**, 184-191 (2016).
13. Thyme, S.B., Akhmetova, L., Montague, T.G., Valen, E. & Schier, A.F. Internal guide RNA interactions interfere with Cas9-mediated cleavage. *Nat Commun* **7**, 11750 (2016).
14. Oakes, B.L., Fellmann, C., Rishi, H., Taylor, K.L., Ren, S.M., Nadler, D.C., Yokoo, R., Arkin, A.P., Doudna, J.A. & Savage, D.F. CRISPR-Cas9 Circular Permutants as Programmable Scaffolds for Genome Modification. *Cell* **176**, 254-267 e16 (2019).
