# Supplementary material for: Controlling CRISPR-Cas9 with ligand-activated and ligand-deactivated sgRNAs
Source: Nat Commun. 2019 May 9;10:2127. doi: 10.1038/s41467-019-09985-2 (PMC6509140; doi:10.1038/s41467-019-09985-2)
Supplement: Supplementary file 3 — Reporting Summary [file 41467_2019_9985_MOESM3_ESM.pdf]

nature research

Corresponding author(s): Tanja Kortemme

Last updated by author(s): Mar 26, 2019

Reporting Summary

Nature Research wishes to improve the reproducibility of the work that we publish. This form provides structure for consistency and transparency in reporting. For further information on Nature Research policies, see Authors & Referees and the Editorial Policy Checklist.

Statistics

For all statistical analyses, confirm that the following items are present in the figure legend, table legend, main text, or Methods section.

n/a	Confirmed	
[ ]	[x]	The exact sample size (*n*) for each experimental group/condition, given as a discrete number and unit of measurement
[ ]	[x]	A statement on whether measurements were taken from distinct samples or whether the same sample was measured repeatedly
[x]	[ ]	The statistical test(s) used AND whether they are one- or two-sided<br>*Only common tests should be described solely by name; describe more complex techniques in the Methods section.*
[x]	[ ]	A description of all covariates tested
[x]	[ ]	A description of any assumptions or corrections, such as tests of normality and adjustment for multiple comparisons
[ ]	[x]	A full description of the statistical parameters including central tendency (e.g. means) or other basic estimates (e.g. regression coefficient) AND variation (e.g. standard deviation) or associated estimates of uncertainty (e.g. confidence intervals)
[x]	[ ]	For null hypothesis testing, the test statistic (e.g. *F*, *t*, *r*) with confidence intervals, effect sizes, degrees of freedom and *P* value noted<br>*Give P values as exact values whenever suitable.*
[x]	[ ]	For Bayesian analysis, information on the choice of priors and Markov chain Monte Carlo settings
[x]	[ ]	For hierarchical and complex designs, identification of the appropriate level for tests and full reporting of outcomes
[x]	[ ]	Estimates of effect sizes (e.g. Cohen's *d*, Pearson's *r*), indicating how they were calculated

Our web collection on statistics for biologists contains articles on many of the points above.

Software and code

Policy information about availability of computer code

Data collection	No software used for data collection.
Data analysis	Analysis scripts are available on GitHub as indicated in the Methods.

For manuscripts utilizing custom algorithms or software that are central to the research but not yet described in published literature, software must be made available to editors/reviewers. We strongly encourage code deposition in a community repository (e.g. GitHub). See the Nature Research guidelines for submitting code & software for further information.

Data

Policy information about availability of data

All manuscripts must include a data availability statement. This statement should provide the following information, where applicable:

- Accession codes, unique identifiers, or web links for publicly available datasets
- A list of figures that have associated raw data
- A description of any restrictions on data availability

Data Availability statement included in the Methods section: All relevant data are reported in the main text or Supplementary Information. The source data underlying Figs 1d and e are provided as a Source Data file.

Field-specific reporting

Please select the one below that is the best fit for your research. If you are not sure, read the appropriate sections before making your selection.

[x] Life sciences [ ] Behavioural & social sciences [ ] Ecological, evolutionary & environmental sciences

Life sciences study design

All studies must disclose on these points even when the disclosure is negative.

Sample size	We did not choose sample sizes to detect pre-specified effect sizes, but performed experimental replicates as indicated for all assays, reported throughout the manuscript (Figures, Methods, Supplementary Tables and Figures).
Data exclusions	All samples from a given experiment (or multiple experiments from different days, as indicated) that were accompanied with appropriate positive and negative controls were included.
Replication	We confirmed reproducibility using replicates as indicated throughout the manuscript. All attempts at replicates were successful.
Randomization	No randomization was used (there were no experimental groups).
Blinding	No blinding was used (there was no group allocation).

Reporting for specific materials, systems and methods

We require information from authors about some types of materials, experimental systems and methods used in many studies. Here, indicate whether each material, system or method listed is relevant to your study. If you are not sure if a list item applies to your research, read the appropriate section before selecting a response.

Materials & experimental systems

n/a	Involved in the study	
☒	☐	Antibodies
☐	☒	Eukaryotic cell lines
☒	☐	Palaeontology
☒	☐	Animals and other organisms
☒	☐	Human research participants
☒	☐	Clinical data

Methods

n/a	Involved in the study	
☒	☐	ChIP-seq
☐	☒	Flow cytometry
☒	☐	MRI-based neuroimaging

Eukaryotic cell lines

Policy information about cell lines

Cell line source(s)	HEK293T (293FT; Thermo Fisher Scientific).
Authentication	None of the cell lines were authenticated.
Mycoplasma contamination	HEK293T and HEK-RT1 cells were tested for absence of mycoplasma contamination (UC Berkeley Cell Culture facility) by fluorescence microscopy of methanol fixed and Hoechst 33258 (Polysciences #09460) stained samples.
Commonly misidentified lines (See ICLAC register)	No commonly misidentified cell lines.

Flow Cytometry

Plots

Confirm that:

☒ The axis labels state the marker and fluorochrome used (e.g. CD4-FITC).

☒ The axis scales are clearly visible. Include numbers along axes only for bottom left plot of group (a 'group' is an analysis of identical markers).

☐ All plots are contour plots with outliers or pseudocolor plots.

☐ A numerical value for number of cells or percentage (with statistics) is provided.

Methodology

Sample preparation	Bacterial, fungal, and mammalian samples were prepared as described in the Methods section.
Instrument	As described in the Methods section, the following instruments were used for flow cytometry: BD LSRII, Attune NxT, BD FACSAria II.

Software	Flow cytometry data were analyzed using scripts made available on GitHub as described in the Methods section.
Cell population abundance	Sorting was used for library screening as described in Figure 2 in the main manuscript. Individual clones from the sorted populations were sequenced and validated in separate experiments including replicates, as shown in the corresponding Figures in the main text and Supplementary Information.
Gating strategy	The gating strategy for library screening is described in the Methods section. Briefly, gates were drawn (if possible) based on the position of a control population, or (otherwise) based on the position of the most extreme library members. The gates typically contained 1%-5% of the total population.

☐ Tick this box to confirm that a figure exemplifying the gating strategy is provided in the Supplementary Information.
