# Supplementary figures and images for: Controlling CRISPR-Cas9 with ligand-activated and ligand-deactivated sgRNAs
Source: Nat Commun. 2019 May 9;10:2127. doi: 10.1038/s41467-019-09985-2 (PMC6509140; doi:10.1038/s41467-019-09985-2)

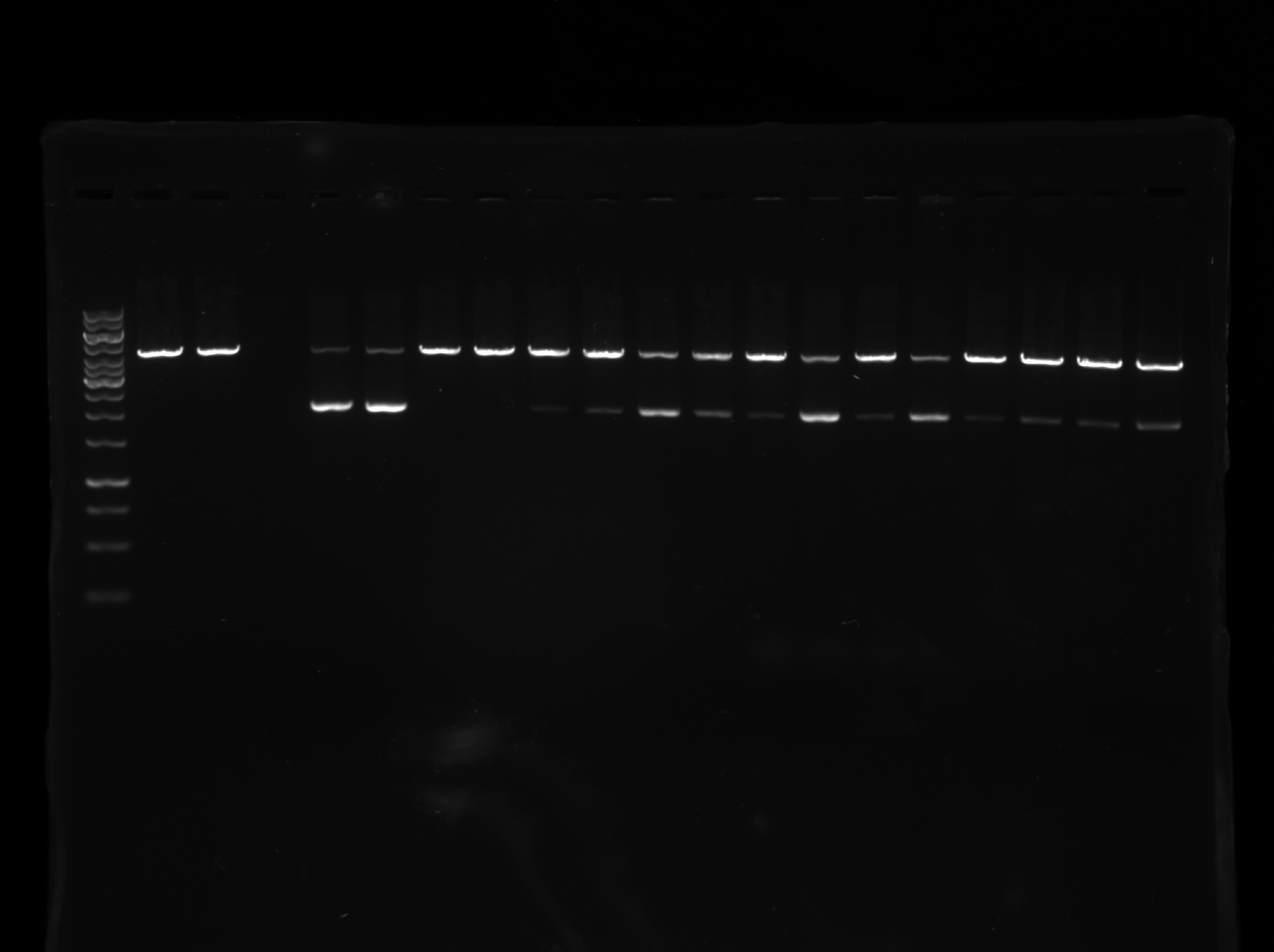

Supplement: Supplementary file 4 — Source Data [file 41467_2019_9985_MOESM4_ESM.zip › NCOMMS-18-14889B source data/raw_data/fig_1d/20160318_repeat_working_designs.tif]

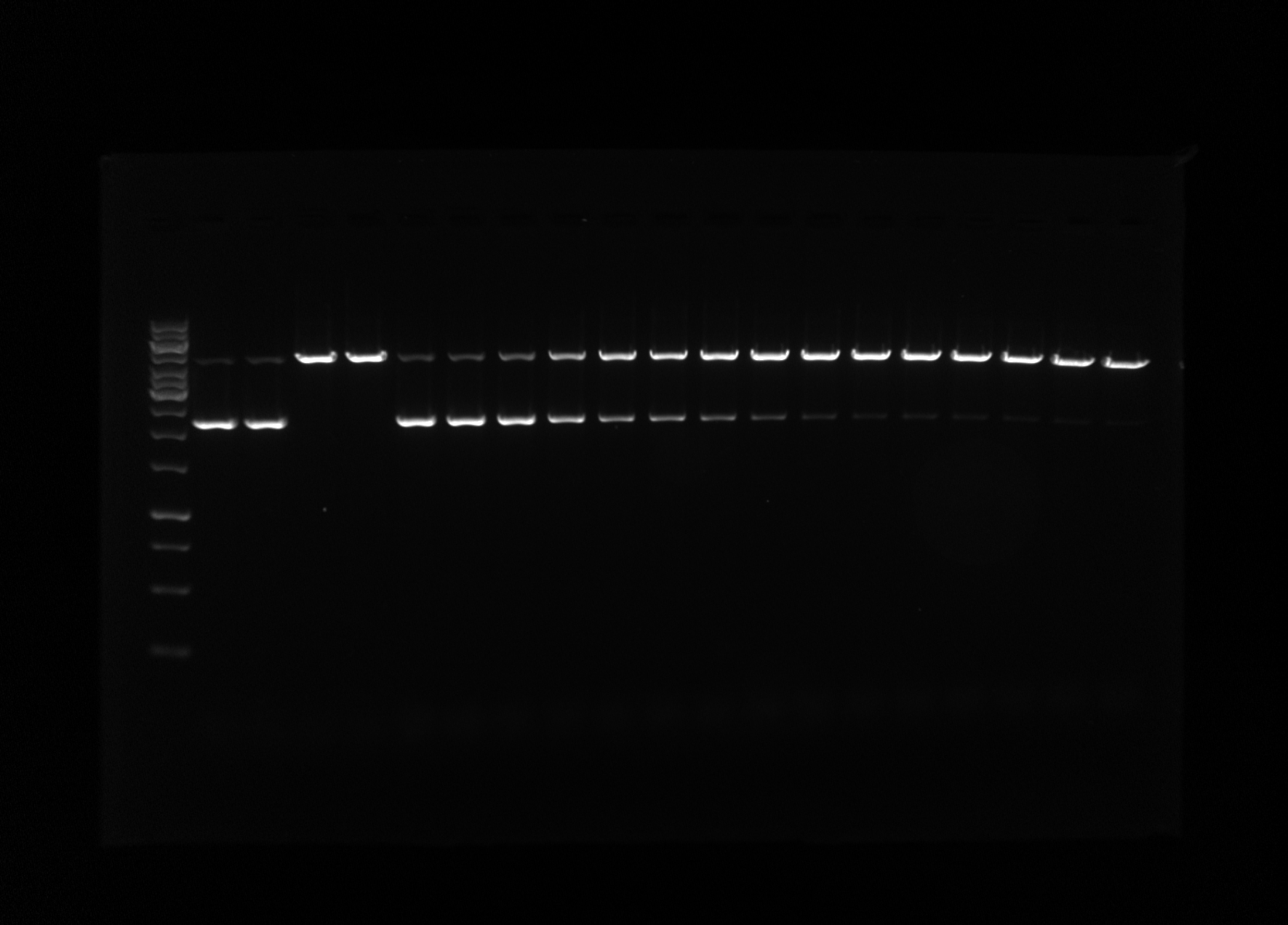

Supplement: Supplementary file 4 — Source Data [file 41467_2019_9985_MOESM4_ESM.zip › NCOMMS-18-14889B source data/raw_data/fig_1e/20171003_titrate_cb_new_rna.tif]
